# Exploring the Rheology and Clinical Potential of Calcium Hydroxylapatite‐Hyaluronic Acid Hybrids

**DOI:** 10.1111/jocd.70473

**Published:** 2025-10-13

**Authors:** Ewelina Kaczuba, Nabil Fakih‐Gomez, Jonathan Kadouch, Rolf Bartsch, Carla Pecora, Yana A. Yutskovskaya, Nadine Hagedorn, Radia El‐Banna, Sarah Backfisch, Alec D. McCarthy

**Affiliations:** ^1^ Filler and Medical Device Development Merz Aesthetics GmbH Frankfurt Germany; ^2^ Department of Facial Plastic & Cranio‐Maxillo‐Facial Surgery Fakih Hospital Khaizaran Lebanon; ^3^ Practice for Aesthetic Dermatology, ReSculpt Clinic Amsterdam the Netherlands; ^4^ Plastic Surgery, TheAesthetics Vienna Austria; ^5^ The Filler Academy, Österreich Vienna Austria; ^6^ Private Practice, Clínica Dermatologie São Paulo Brazil; ^7^ Dermatovenerology and Cosmetology Department Pacific State Medical University of Health Moscow Russia; ^8^ Global Medical Affairs Merz Aesthetics Raleigh North Carolina USA

**Keywords:** Belotero, CaHA, CaHA‐CMC, calcium hydroxylapatite, CPM, hybrid filler, rheology

## Abstract

**Background:**

Combining calcium hydroxylapatite‐carboxymethylcellulose (CaHA‐CMC) with hyaluronic acid (HA) products using Cohesive Polydensified Matrix (CPM) technology leverages the volumizing and hydrating effects of HA and draws upon the regenerative properties of CaHA‐CMC, providing a potentially synergistic approach in aesthetic filling treatments. Despite this potential, the rheological and physical properties of these hybrid fillers remain unreported.

**Objective:**

To characterize the rheological properties of CaHA‐CMC blended with various CPM‐HA products and identify factors influencing their physical and clinical behavior.

**Methods:**

Hybrid fillers were prepared by mixing CaHA‐CMC with different CPM‐HA products at varying ratios (1:1 to 1:4 syringe CaHA:syringe CPM). Rheological properties, including storage modulus (*G*′), loss modulus (*G*″), complex shear modulus (G*), tan delta (tan *δ*), and complex viscosity (η*), were measured using an oscillatory rheometer. Extrusion force, cohesivity, stability, and axial strain (*ε*_*a*_) were also evaluated.

**Results:**

The rheological properties of the hybrid fillers varied with the type of HA, mixing ratio, and crosslinking degree. Higher HA concentrations increased *G*′, *G*″, and η*, enhancing gel stiffness and resistance to deformation, while increased HA dilution and lower crosslinking favored spread and injectability. The addition of CaHA‐CMC to CPM‐HA products always increased the *G*′ in a volume‐dependent manner. These properties were closely correlated with the fillers' performance in different tissue planes.

**Conclusions:**

The properties of CaHA‐CMC and CPM‐HA hybrids influence their injection behavior and clinical outcomes. Understanding these factors can guide filler selection and application techniques to maximize safety, efficacy, and patient satisfaction in aesthetic treatments.

## 1 Introduction

In recent years, combining calcium hydroxylapatite‐carboxymethylcellulose (CaHA‐CMC; Radiesse, Merz North America Inc., Franksville, WI) with hyaluronic acid (HA) products, specifically those with Cohesive Polydensified Matrix technology (CPM; Belotero, Anteis S.A., Plan‐les‐Ouates, Switzerland, a company of the Merz Aesthetics group), has become increasingly popular in aesthetic medicine [1, 2, 3, 4, 5, 6]. These blends are designed to take advantage of the unique properties of each component: the immediate volumizing and hydrating effects of HA and the biostimulatory properties of CaHA‐CMC. By hybridizing CaHA‐CMC and CPM‐HA gels, practitioners generally aim to achieve a regenerative filler that provides a long‐term, direct filling effect with HA while simultaneously promoting skin extracellular matrix (ECM) regeneration induced by CaHA microsphere–fibroblast interactions [7, 8].

HA is a naturally occurring polysaccharide found in the ECM of the skin, where it plays a critical role in maintaining hydration and tissue integrity [9]. In its cross‐linked form, HA is widely used in dermal fillers to provide immediate volume, structure, and hydration. The Belotero line utilizes CPM technology, which uniquely involves dual‐phase crosslinking, to create monophasic polydensified HA gels. The varied densities within the CPM‐HA gel architecture help to optimize tissue integration and reduce the risk of migration, thereby creating a natural‐looking, uniform appearance post‐injection [10, 11]. Belotero Revive (CPM‐R) combines HA (at 20 mg/mL) with glycerol (at 17.5 mg/mL) and is delivered superficially to enhance skin hydration and dermal quality. Belotero Volume (CPM‐V) and Intense (CPM‐I) have higher HA concentrations and are delivered to deeper planes where they offer greater volumization and projection. This versatility allows practitioners to select the most appropriate CPM‐HA product for different clinical objectives, complementing CaHA in hybrid filler formulations with the goal of maximizing immediate and long‐term aesthetic outcomes.

CaHA‐CMC is an FDA‐approved dermal filler and regenerative biostimulator that is composed of 70% v/v CMC and 30% v/v CaHA microspheres (25–45 μm in diameter). When undiluted or hypodiluted (1:< 1), CaHA‐CMC acts as both a dermal filler and regenerative biostimulator and provides contouring and volumization [12]. When diluted (1:1) or hyperdiluted (1:> 1), the direct volumizing effect of CaHA‐CMC is diminished due to the miscibility of CMC in aqueous dilutions, and the biostimulation persists as the primary aesthetic mechanism of action [13]. The stimulation of primary ECM constituents is theorized to result in long‐term tissue remodeling and skin quality improvements associated with such treatments [14, 15, 16]. HA alone retains primarily a direct filling effect that degrades non‐linearly. When combined, CaHA‐CMC and HA blends may provide immediate volumization and smoothing along with gradual biostimulation. It is theorized that, mechanistically, as the HA component degrades over time, it creates a gradual transition where cells increasingly interact with the calcium hydroxylapatite (CaHA) microspheres. The increased CaHA‐fibroblast interaction promotes neocollagenesis, which leads to sustained tissue tightening and a prolonged aesthetic correction, even after the initial volumizing effect of the HA diminishes [17, 18]. This dual‐action mechanism makes CaHA‐CMC and HA (CaHA‐HA) hybrids particularly appealing for patients seeking both immediate enhancement and enduring improvements in skin quality.

Understanding the rheological properties of these hybrid fillers is essential for optimizing clinical performance, injection planes, and treatment areas [19, 20, 21]. The viscoelastic properties dictate the filler's ability to directly fill, spread, integrate, and maintain contour within tissues while avoiding complications like migration or lump formation. Further, different rheological properties have unique physico‐clinical correlates (Table 1). The specific characteristics of CaHA‐HA blends, including the ratio of CaHA to HA, the degree of HA crosslinking, and the particle concentration of the CaHA microspheres, can significantly impact their behavior upon injection and over time. However, these properties have not been thoroughly investigated, leaving a gap in our knowledge regarding their best applications and potential risks.

**TABLE 1:** Rheological and physical measurements, symbols, descriptions, and physico‐clinical correlations. Table adapted with permission from McCarthy and Soares et al. [13].

Measure	Symbol	Physical description	Physico‐clinical correlate
Elastic (storage) modulus	*G*′	Ability to store energy elastically and resist shear deformation	*Gel stiffness*. A high *G*′ equates with a stronger gel that can project tissues and retain its implanted shape
Viscous (loss) modulus	*G*″	Ability to dissipate energy through shear plastic deformation	*Gel pliability*. A high *G*″ indicates the gel is losing strength at an increasing rate, but is meaningful only when compared to *G*′
Complex shear modulus	G*	Combined measurement of the elastic and viscous properties (G* = √(*G*′^2^ + *G*″^2^))	*Overall gel behavior*. Represents the total resistance of a gel to deformation, combining both its elastic and viscous components
Tan delta	Tan(*δ*) (*G*″/*G*′)	Viscoelastic character of a gel based on the ratio of the viscous to elastic modulus. Colloids with tan *δ* > 1 are more viscous than elastic, behaving more like fluids. Colloids with tan *δ* < 1 are more elastic than viscous, behaving more like solids	*Gel fluidity*. Gels with high tan *δ* feel thinner to the touch. In contrast, gels with a low tan *δ* feel thicker or stronger
Cohesivity		Ability to resist fragmentation when exposed to shear, tensile, or compressive forces and strains	*Gel stickiness*. A highly cohesive gel tends to stick together, resisting disintegration and dispersal
Complex viscosity	η*	Ability to resist flow in response to change in strain/flow rate	*Fluid thickness*. A fluid with a higher complex viscosity (e.g., honey) feels thicker than a fluid with a low complex viscosity (e.g., olive oil)
Extrusion force		Force required to push or squeeze a gel through an opening	*Injection resistance*. A gel with a high extrusion force requires greater syringe pressure to inject
Axial strain	*ε*_*a*_	Change in gel's height after compression computed as: *ε*_*a*_ = Change in Height/Original Height	*Gel projection and spread*. A gel with a lower axial strain maintains its structure under compressive loads and generally spreads less than gels with higher axial strains

Therefore, this study aims to characterize the rheological and physical properties of CaHA‐HA blended with various CPM products. In addition, this exploratory analysis also aims to elucidate the factors that contribute to the physical properties of the hybrid CaHA‐HA fillers. The findings will offer guidance on optimizing filler selection and application techniques to potentially maximize both the immediate and long‐term benefits of CaHA‐HA hybrids in aesthetic treatments.

## 2 Materials and Methods

### 2.1 Materials

CaHA‐CMC was obtained from Merz North America Inc. (Franksville, Wisconsin, USA). The HA gels (CPM‐HA) studied include Belotero Volume (CPM‐V), Belotero Revive (CPM‐R), Belotero Balance (CPM‐B), and Belotero Intense (CPM‐I), and were manufactured by Anteis S.A. (Plan‐les‐Ouates, Switzerland, a company of the Merz Aesthetics group). These products contained 0.3% lidocaine, except for CPM‐R, which is not available with lidocaine. Terumo syringes (3 mL Luer Lock, Terumo Europe N.V.), Baxter RAPIDFILL connectors (Baxter Healthcare, USA), BD syringes (3 mL, 5 mL, and 10 mL Luer Lock, Becton, Dickinson and Company, USA), BBraun Injekt Luer Lock syringes (2 mL, BBraun Melsungen AG, Germany), and TSK 27G ½″ (TSK Laboratory International, Tochigi‐Ken, Japan) were used for sample preparation and testing. The rheological measurements were conducted using an Anton Paar oscillatory rheometer (MCR 302e, Anton Paar GmbH, Graz, Austria), and texture analysis was performed using a texture analyzer (TA.XTplusC Texture Analyzer, Stable Micro Systems, Surrey, United Kingdom).

### 2.2 Hybrid Filler Sample Preparation

**FIGURE 1:**
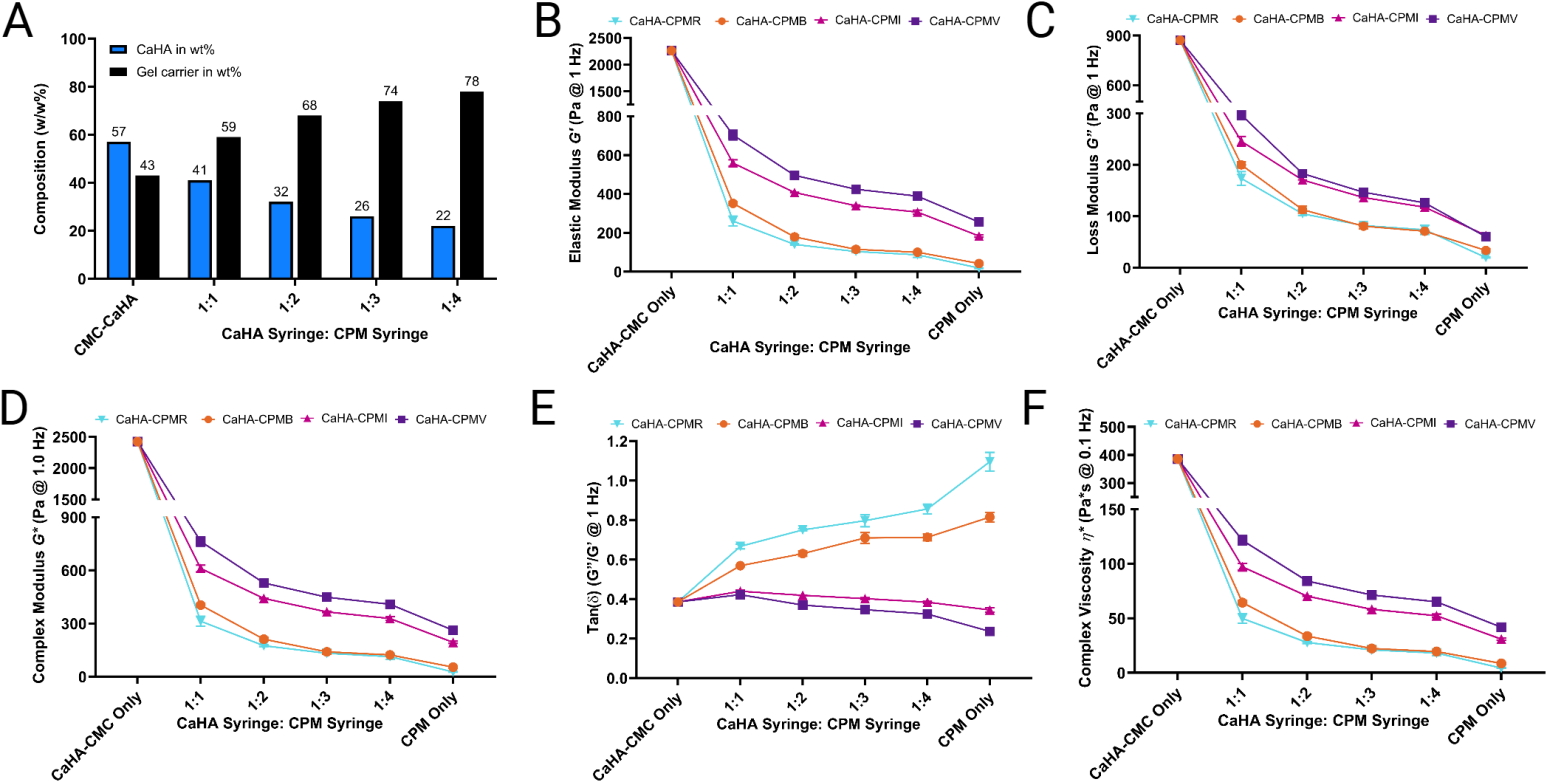
Composition and rheological properties of hybrid CaHA‐CPM fillers. (A) CaHA microsphere and gel carrier composition at each dilution. (B) Elastic moduli *G*′, (C) loss moduli *G*″, (D) complex moduli G*, (E) tan(*δ*), and (F) complex viscosity (η*) of CaHA‐CMC only, CaHA‐CPM 1:1–1:4, and CPM only measured at 1 Hz.

Hybrid fillers were prepared by combining whole syringes of CaHA‐CMC (1.5 mL) with varying amounts of CPM‐HA gels using a Luer lock‐to‐Luer lock connector (RAPIDFILL, Baxter Healthcare, USA). The HA gels were drawn into separate mixing syringes and combined with the CMC‐CaHA by alternately depressing the plungers for a defined number of mixing strokes: 15 for CPM‐V, 30 for CPM‐I and CPM‐B, and 40 for CPM‐R. The mixing ratios of CaHA‐CMC to CPM‐HA varied from 1:1 to 1:4 (syringe CaHA‐CMC: syringe CPM‐HA) (Table 2). The number of mixing strokes required to achieve a homogeneous distribution depended on the type of CPM‐HA and ranged from 15 to 40, based on prior evaluations described in Fakih Gomez et al. [4, 22] The final composition (w/w%) of CaHA microspheres and gel carrier (either CMC or CPM‐HA) for each hybrid is listed in Table S1 and shown in Figure 1A.

**TABLE 2:** Hybridization dilution table used in this study.

CaHA‐CMC to CPM‐HA ratio (syringe: syringe)	Amount of CaHA‐CMC		Amount of CPM‐HA	
	Syringes	Volume (mL)	Syringes	Volume (mL)
0:1	0	0.00	1	1.00
1:1	1	1.50	1	1.00
1:2	1	1.50	2	2.00
1:3	1	1.50	3	3.00
1:4	1	1.50	4	4.00

### 2.3 Rheological Measurements

Rheological properties, including the storage modulus (*G*′), loss modulus (*G*″), complex shear modulus (complex modulus, G*), tan(*δ*), and complex viscosity (η*), were measured in triplicate using the Anton Paar oscillatory rheometer. The tests were conducted at a constant shear deformation of 0.1% over a frequency range of 10 to 0.1 Hz, with measurements evaluated at 1 Hz. Testing conditions were maintained at 25°C with a PP20 measurement system. All samples were measured immediately after preparation and after 24 h of storage at room temperature to assess stability over time.

### 2.4 Extrusion Force Measurements

Extrusion force was evaluated using a TA.XTPLUS Texture Analyzer. Pure and hybrid fillers were transferred into a CaHA‐CMC syringe (1.5 mL) BD glass syringes after mixing, and the extrusion force was measured through a TSK 27G ½″ thin‐wall needle. Force measurements for neat fillers were taken directly from their original syringes. The force was recorded at specific intervals using the compression mode of the texture analyzer, and average values were calculated from triplicate measurements.

### 2.5 Cohesivity Measurements

Cohesivity was assessed using drop weight testing with a TA.XTPLUS Texture Analyzer equipped with an 18G 1½″ Terumo needle. Drop weight testing, when applied within HA gels with common crosslinking technology, can effectively measure cohesion [13, 23]. The drop weight test was performed five times (10 drops each) for each hybrid and non‐diluted CaHA‐CMC at a fixed extrusion rate of 7.8 mm/min, with results expressed in μL per drop to account for density differences between the CaHA‐CMC and CPM‐HA hybrids as previously established when measuring drop weight values for CaHA‐containing samples [13].

### 2.6 Axial Strain Measurements

Axial Strain (*ε*_*a*_) was determined by compressing a 2 mL sample with a PP40 plate‐plate system on a rheometer, applying a constant force of 1.0 N for 120 s at 25°C based on similar experimental designs [24]. The maximum gap size after 105 s was recorded to determine the change in height, which was used to calculate *ε*_*a*_.

### 2.7 Sedimentation Stability

Sedimentation stability was evaluated by transferring the hybrid filler samples into the mixing syringes immediately after mixing. The syringes were then stored upright at room temperature for two‐time intervals: 0.5 h and 24 h. After the specified incubation periods, the contents of each syringe were extruded in two portions (front and back) into separate ceramic dishes for analysis. The solid content (%) of each portion was determined by ashing the samples at increasing temperatures (from room temperature up to 750°C) to eliminate organic compounds, leaving only CaHA particles. The remaining material was weighed to calculate the % solids. This process was repeated in triplicate for each sample. The maximum differences in solid content between the front and back portions were recorded for each time point and compared to the initial homogeneity values measured directly after mixing. The sedimentation stability of the samples was determined by evaluating the consistency of solid content values over time.

### 2.8 Evaluation of Biological Effects

To assess the biological effects of hybrids, a series of secondary analyses of previously published works were conducted. In the case of Bravo et al. (2022) and Bravo et al. (2024), WebPlotDigitizer, a web‐based application for accurately extracting data from graphs, was used to extract raw dermal thickness values at each time point for CaHA‐CMC (1:1) and CaHA‐CPM‐I (1.5 mL: 2.0 mL) which were subsequently plotted together [25, 26, 27]. In both cases, the investigators used a linear 18 MHz transducer high‐frequency ultrasound LogicE device (GE Healthcare) with a high‐frequency linear probe (L8‐L18i‐RS) for their measurements. To measure the effect of hybrids on neocollagenesis, histological samples were supplied by Y. Yutskovskaya based on a previous publication [8]. Yutskovskaya et al. conducted an observational, comparative study in healthy female subjects receiving (1:1 syringe: syringe) CaHA‐CPM injections. Biopsies were stained with Van Gieson's Picrofuchsin (VGP), which stains collagen red/pink. For our analysis, VGP‐stained samples at baseline, 1 month, and 4 months underwent color deconvolution along the H DAB preset color split on ImageJ based on previous protocols [28, 29]. A threshold was automatically applied on the blue/purple channel split to highlight collagen‐positive areas. Ten random regions of interest were selected using the same pixel area and the average collagen‐expressing areas were recorded and graphed.

### 2.9 Statistical Analysis

All data are presented as mean ± standard deviation, where applicable. No outliers were excluded from any of the statistical analyses across all tests. For comparisons of single‐group averages, type I, fixed‐effect, one‐way ANOVAs were utilized, followed by Tukey's multiple comparisons post hoc test to determine the significance of differences between group means. In cases of grouped data, a two‐way ANOVA was performed, and mean values were compared both between and within groups using Tukey's and/or Sidak's post hoc test for significance. Significance levels are indicated graphically as follows: ns/no graphic for *p* > 0.05 (not significant), *p < 0.05, **p < 0.01, ***p < 0.001, and ****p < 0.0001. All statistical analyses and graphing were conducted using GraphPad Prism Volume 10.2 (GraphPad, San Diego, CA, USA), while all images and figures were created using BioRender (Toronto, Ontario, Canada). Given the number of pairwise comparisons on each graph, summary values are given in the Tables S2–S16.

## 3 Results

### 3.1 Rheological Properties

The rheological properties of the hybrid fillers demonstrated significant variations based on the CPM‐HA utilized and the dilution ratio employed. As detailed in Table 3, *G*′, *G*″, G*, tan(*δ*), and η* varied significantly, but followed generalized trends. Among the different CPM‐HAs, CPM‐V hybrids exhibited the highest *G*′ values followed by CPM‐I hybrids, CPM‐B hybrids, and CPM‐R hybrids across all dilution ratios (Figure 1). *G*′ values for each hybrid followed a similar pattern consisting of a significant strengthening effect with the addition of CaHA‐CMC that diminished with increasing CPM‐HA dilution volumes (Figure 1B). Regardless of the CPM‐HA dilution volume, no hybrids had *G*′ values that returned to the *G*′ values of their neat CPM‐HA component.

**TABLE 3:** Summary of rheometric and physical properties of neat and hybrid fillers evaluated in this study.

Number of syringes		Elastic modulus G′ (Pa)	Viscous modulus G″ (Pa)	Complex shear modulus G* (Pa)	tan(*δ*)	Complex viscosity η* (Pa·s)	Cohesivity (μL/drop)	Extrusion force (N)
*CaHA‐CMC*	*CPM‐R*							
0	1	18.15 ± 1.59	19.84 ± 1.02	26.90 ± 1.80	1.10 ± 0.05	4.28 ± 0.29	51.13 ± 4.63	6.69 ± 0.29
1	1	260.58 ± 24.95	173.52 ± 13.43	313.08 ± 28.21	0.67 ± 0.01	49.83 ± 4.50	22.67 ± 1.80	11.47 ± 1.98
1	2	139.81 ± 4.60	104.90 ± 3.14	174.79 ± 5.54	0.75 ± 0.01	27.82 ± 0.88	22.56 ± 1.15	10.23 ± 0.55
1	3	103.08 ± 5.98	82.03 ± 1.98	131.75 ± 5.80	0.80 ± 0.03	20.97 ± 0.93	25.33 ± 0.41	9.17 ± 0.09
1	4	86.31 ± 13.05	73.69 ± 8.92	113.50 ± 15.72	0.86 ± 0.03	18.06 ± 2.50	25.74 ± 1.30	8.66 ± 0.23
*CaHA‐CMC*	*CPM‐B*							
0	1	41.54 ± 5.07	33.77 ± 3.17	53.54 ± 5.93	0.82 ± 0.02	8.52 ± 0.94	54.83 ± 1.39	7.94 ± 1.62
1	1	351.23 ± 9.28	199.86 ± 5.88	404.12 ± 10.94	0.57 ± 0.00	64.32 ± 1.74	25.10 ± 0.58	15.63 ± 3.40
1	2	178.91 ± 9.19	112.85 ± 6.70	211.53 ± 11.18	0.63 ± 0.01	33.67 ± 1.78	27.74 ± 0.96	13.39 ± 0.46
1	3	114.31 ± 11.21	80.91 ± 5.48	140.07 ± 12.22	0.71 ± 0.03	22.29 ± 1.95	27.75 ± 1.04	12.22 ± 0.94
1	4	99.83 ± 1.82	71.16 ± 0.69	122.60 ± 1.31	0.71 ± 0.02	19.52 ± 0.21	28.43 ± 1.21	12.51 ± 0.87
*CaHA‐CMC*	*CPM‐I*							
0	1	183.26 ± 6.46	63.12 ± 0.69	193.83 ± 6.17	0.34 ± 0.01	30.85 ± 0.98	35.70 ± 2.19	19.68 ± 0.26
1	1	558.88 ± 16.89	245.75 ± 9.45	610.53 ± 19.10	0.44 ± 0.01	97.18 ± 3.04	29.38 ± 0.92	16.95 ± 2.11
1	2	407.29 ± 1.37	170.65 ± 0.17	441.60 ± 1.33	0.42 ± 0.00	70.28 ± 0.22	27.08 ± 0.74	16.69 ± 2.16
1	3	339.26 ± 4.08	136.48 ± 0.35	365.69 ± 3.82	0.40 ± 0.00	58.20 ± 0.61	26.45 ± 1.64	15.82 ± 0.97
1	4	306.53 ± 9.90	117.68 ± 2.36	328.34 ± 10.07	0.38 ± 0.01	52.26 ± 1.60	26.22 ± 0.43	16.22 ± 0.99
*CaHA‐CMC*	*CPM‐V*							
0	1	255.2 ± 2.62	60.31 ± 0.81	262.23 ± 2.73	0.24 ± 0.00	41.74 ± 0.43	19.40 ± 0.61	8.72 ± 0.29
1	1	703.22 ± 25.23	296.94 ± 9.01	763.35 ± 26.59	0.42 ± 0.01	121.49 ± 4.24	19.16 ± 1.66	13.47 ± 1.31
1	2	495.83 ± 1.62	183.13 ± 4.84	528.58 ± 2.75	0.37 ± 0.01	84.12 ± 0.44	20.18 ± 0.99	13.77 ± 0.58
1	3	424.12 ± 4.04	146.76 ± 4.86	448.80 ± 5.00	0.35 ± 0.01	71.42 ± 0.80	19.37 ± 1.20	13.82 ± 1.28
1	4	388.95 ± 2.48	126.16 ± 2.86	408.90 ± 3.10	0.32 ± 0.01	65.08 ± 0.50	19.61 ± 0.70	13.10 ± 1.08

Similarly, the viscous modulus (*G*″) followed a comparable pattern, where CPM‐V hybrids demonstrated the highest *G*″ values followed by CPM‐I, CPM‐B, and CPM‐R at a 1:1 dilution (Figure 1C). At 1:3 and 1:4 dilutions, CPM‐B and CPM‐R had similar *G*″ values. Each hybrids *G*″ values diminished progressively with increased dilution ratios, aligning with the observed decrease in *G*′.

The complex modulus (G*), representing the combined elastic and viscous properties, mirrored the trends seen in *G*′ and *G*″ (Figure 1D). CPM‐V hybrids at a 1:1 dilution exhibited the highest G* of 763.35 Pa, followed by CPM‐I, CPM‐B, and CPM‐R. Higher dilution ratios resulted in lower G* values across all CPM‐HA types, indicating a reduction in the overall resistance of the gel to deformation. Notably, CPM‐B and CPM‐R did not significantly differ at 1:3 and 1:4 dilutions.

Tan(*δ*) values provided insights into the fluidity of the gels and did not follow a uniform pattern across each hybrid (Figure 1E). For the predominantly viscous CPM‐HA fillers, CPM‐R and CPM‐B, the tan(*δ*) values decreased significantly. In other words, the addition of CaHA‐CMC to CPM‐R and CPM‐B decreased fluidity. Specifically, in the case of CPM‐R, the tan(*δ*) decreased from 1.1 (neat) to 0.67 at a 1:1 dilution volume and stayed under 1.0 out to a 1:4 dilution (tan(*δ*) = 0.86). Thus, CPM‐R on its own has predominantly fluid‐like behavior, which transitions to predominantly solid‐like behavior with the addition of CaHA‐CMC. In contrast, CPM‐I and CPM‐V had increased tan(*δ*) values at each dilution relative to their neat forms, indicating an increase in fluidity despite then increase in *G*′. CaHA‐CMC has a higher tan(*δ*) than CPM‐I and CPM‐V, thus the trend with tan(*δ*) is gravitation towards the tan(*δ*) of CaHA‐CMC.

Complex viscosity (η*) measurements also mirrored the trend observed with *G*′, with CPM‐V having the higher complex viscosity, followed by CPM‐I, CPM‐B, and CPM‐R (Figure 1F). There was a consistent decrease in η* with increasing dilution ratios across all CPM‐HA types, reflecting reduced fluid thickness.

### 3.2 Physical Properties

**FIGURE 2:**
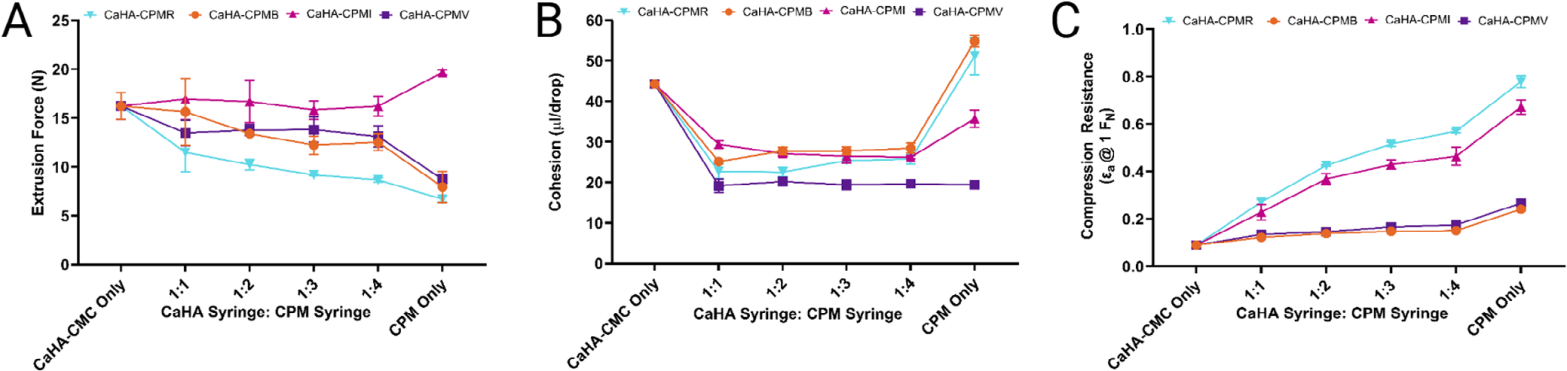
(A) Extrusion force, (B) cohesion, and (C) axial strain for CaHA‐CMC, CaHA‐CPM 1:1–1:4, and CPM only.

Extrusion force measurements did not follow uniform patterning. CPM‐I hybrids required less extrusion force at each dilution compared to neat CPM‐I. In the case of CPM‐V, CPM‐R, and CPM‐B extrusion forces increased with the addition of CaHA‐CMC but uniformly decreased with increasing dilution volume (Figure 2A). Tan(*δ*) was most (negatively) correlated to extrusion force; higher tan(*δ*) generally required less extrusion force.

The cohesivity of the hybrid fillers varied depending on the CPM‐HA type and dilution ratio (Figure 2B). CPM‐I and CPM‐B hybrids maintained higher cohesivity compared to CPM‐V and CPM‐R across all dilution ratios. Although higher dilution ratios generally led to a slight decrease in cohesivity, the impact was more pronounced in CPM‐V and CPM‐R formulations compared to CPM‐I and CPM‐B. The cohesivity between CPM‐V and CaHA‐CPM‐V did not differ significantly.

Axial strain testing revealed that CaHA‐CPM‐I and CaHA‐CPM‐V had similar compression resistance and were significantly stronger than CaHA‐CPM‐B and CaHA‐CPM‐R hybrids (Figure 2C). Additionally, dilutions with increasing volumes of CPM‐HA increased the axial strain with CaHA‐CPM‐B and CaHA‐CPM‐R being most affected by increasing CPM‐HA volume.

**FIGURE 3:**
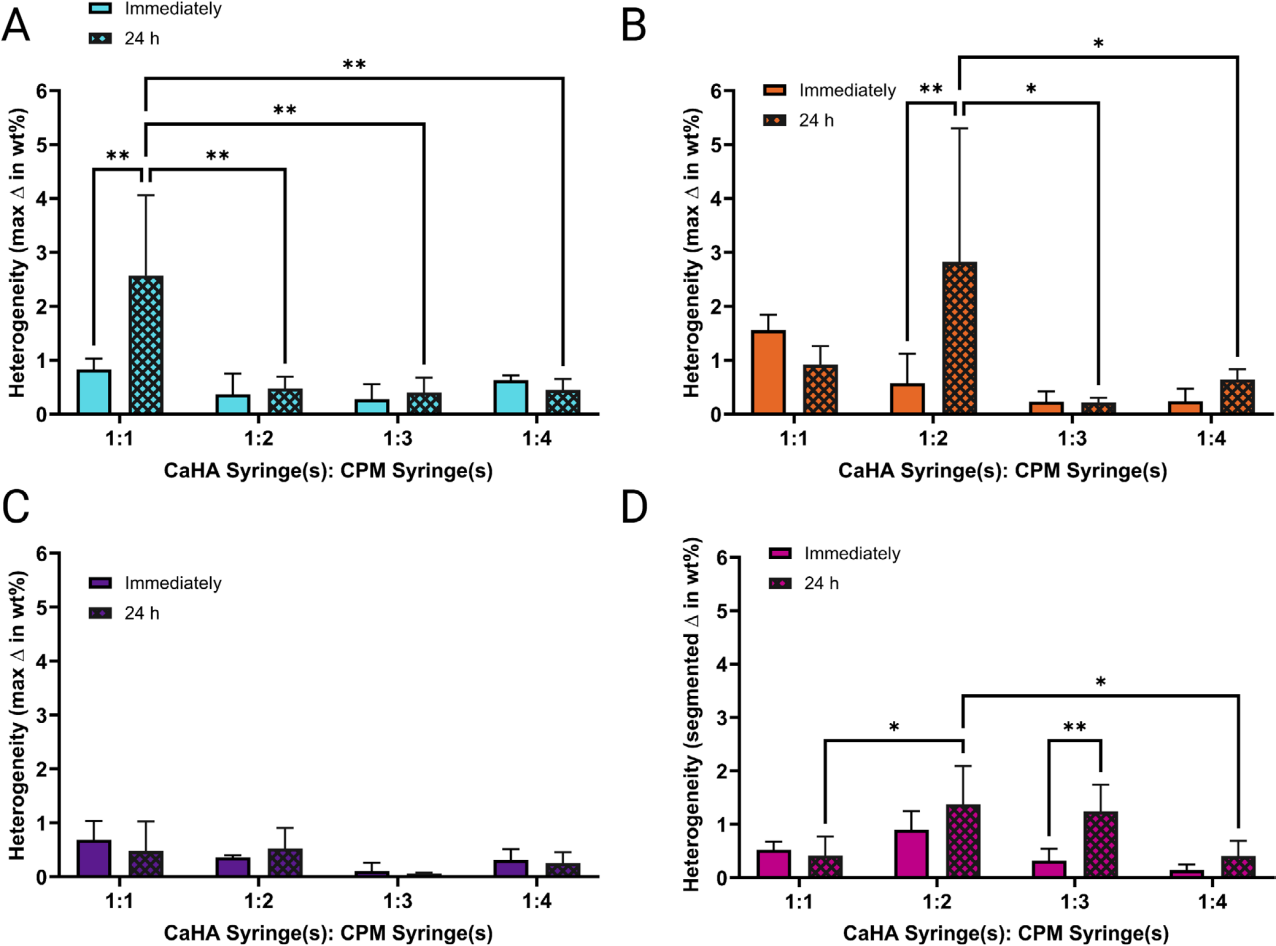
Stability evaluation of different CaHA‐CPM hybrids. Heterogeneity of (A) CaHA‐CPM‐R, (B) CaHA‐CPM‐B, (C) CaHA‐CPM‐V, and (D) CaHA‐CPM‐I 30 min and 24 h after mixing.

During stability evaluation, no visible sedimentation of CaHA particles was observed in any hybrid at 0.5 h or 24 h after mixing. All mixtures at both observation points were homogenous and no measurable sedimentation was found in any of the hybridized CaHA‐CPM fillers. To measure sedimentation, the max deviation in wt% was calculated throughout the filler column in the syringe (Figure 3). While significant differences in heterogeneity existed between dilutions and time points in some instances, the overall max deviation was extremely low, with no hybrid at any timepoint surpassing an average max deviation of more than 3%. In general, hybrids with CPM‐V and CPM‐I had the lowest degree of sedimentation.

## 4 Discussion

### 4.1 Physical Properties

The rheological analysis revealed that the type of CPM‐HA significantly affects the *G*′, *G*″, G*, tan(*δ*), and η* of the hybrid fillers. Specifically, CPM‐V and CPM‐I hybrids consistently exhibited the highest *G*′, *G*″, and η* values across all dilution ratios, indicating superior gel stiffness and resistance to shear stress. This is particularly relevant for applications requiring substantial volumization and structural support, such as deep tissue augmentation. The high *G*′ and low tan(*δ*) values of CPM‐V and CPM‐I hybrids suggest that these formulations can maintain their shape and projection post‐injection, aligning with their intended use for contouring and volumizing.

Conversely, CPM‐B and CPM‐R hybrids exhibited lower *G*′ values and higher tan(*δ*), suggesting less direct volumization and increased spread, making them ideal for soft filling and biostimulation. All hybrid formulations had relatively unchanged cohesivity and extrusion force values that varied more by the base CPM‐HA and less by the dilution ratio. Hybrids generally showed excellent homogeneity, with less than 3% difference in weight distribution through the syringes, even 24 h after mixing. Notably, it may be ideal to inject hybrids, particularly CPM‐R and CPM‐B, immediately after mixing as their homogeneity decreased after 24 h, suggesting moderate sedimentation.

### 4.2 Strengthening Mechanism

The incorporation of CaHA‐CMC into CPM‐HA fillers significantly enhances gel strength, as evidenced by the increased *G*′ and G* values observed in the hybrid formulations. This enhancement can be attributed to the reinforcing interaction between CaHA microspheres and the HA matrix. Analogous to concrete blends, where the addition of aggregates improves the overall structural integrity, the CaHA microspheres act as reinforcing agents within the HA gel network [30]. The rigid CaHA particles provide additional resistance to deformation, thereby increasing the storage and loss moduli of the hybrid filler. Unlike chemical crosslinking agents, CaHA does not facilitate chemical bonds within the HA matrix. Instead, the presence of CaHA microspheres creates an interconnected physical lattice that enhances the overall mechanical properties of the hybrid filler. This physical reinforcement not only improves gel strength but may also contribute to the longevity of the filling effect.

### 4.3 Effects on Biostimulation

**FIGURE 4:**
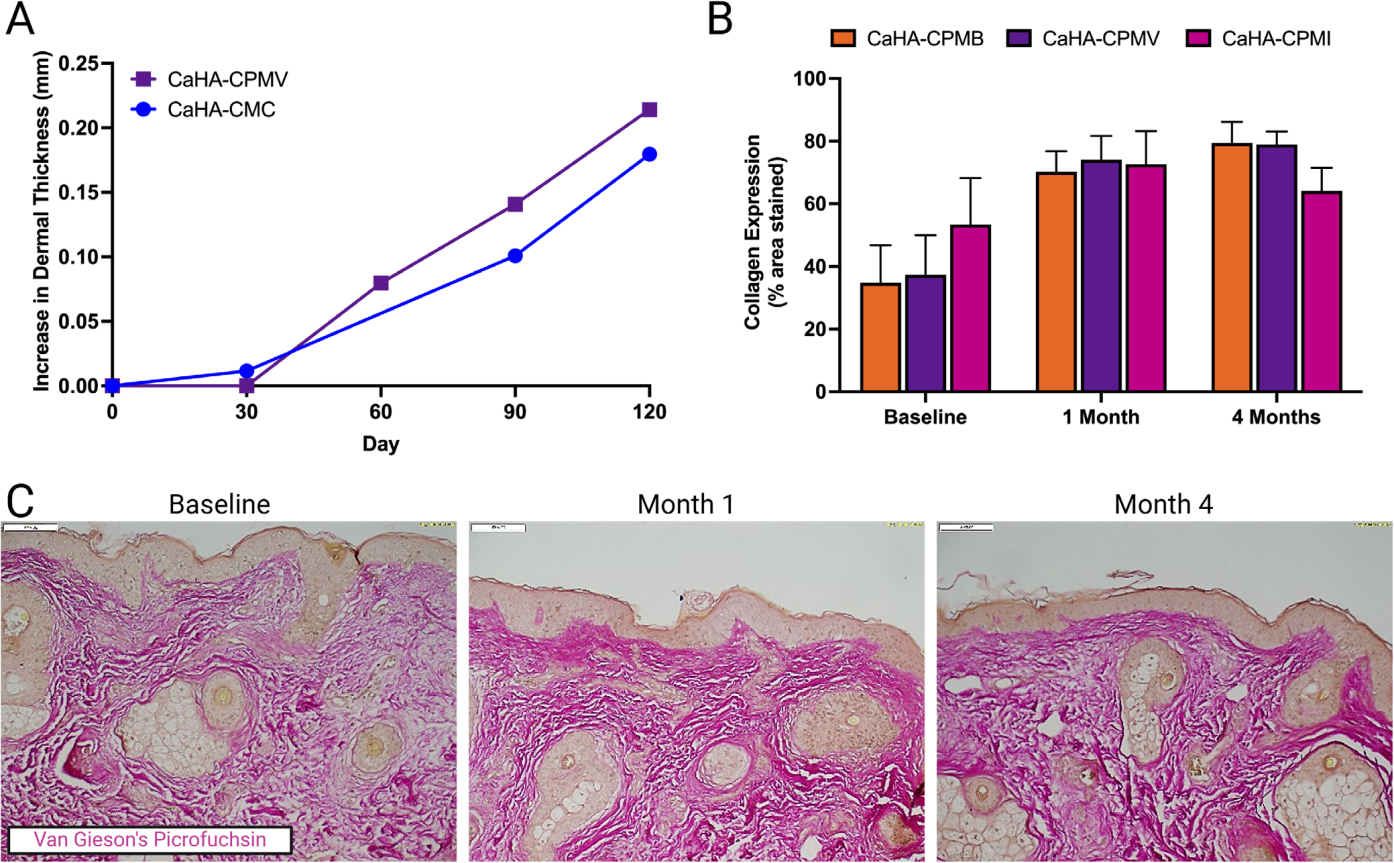
Biological effect of CaHA‐CPM on dermal thickness and collagen expression. (A) Increases in dermal thickness in CaHA‐CMC and CaHA‐CPM‐V, adapted from Bravo et al. and Bravo et al. [25, 26] (B) Changes in collagen expression in tissue treated by CaHA‐CPM‐B, CaHA‐CPM‐V, and CaHA‐CPM‐I and (C) corresponding Van Gieson's Picrofuchsin stains at baseline, 1 month, and 4 months post treatment with CaHA‐CPM‐B adapted with permission from Yutskovskaya et al. [7]

Though no comparative studies have been conducted on the biostimulatory potential of all the hybrids described in this work, previous literature supports the biostimulatory potential of CaHA‐CPM hybrids. Two separate studies by Bravo et al. tracked the dermal thickness of patients treated with CaHA‐CMC and CaHA‐CPM‐I over time using high‐frequency ultrasonography for 120 and 180 days, respectively (Figure 4A) [25, 26]. Both studies demonstrated significant increase in dermal thickness the treatment arms, though the pattern of this increase differed between groups. In the case of 1:1 diluted CaHA‐CMC monotherapy, an exponential increase in dermal thickness was observed through 120 days, while the CaHA‐CPM‐I hybrid group exhibited a more linear increase over the same period. Notably, the CaHA‐CPM‐I group achieved the highest overall percent change in dermal thickness. In another study by Yutskovskaya et al., which was objectively quantified in this study using a previously established method, showed improvements in collagen stimulation when analyzing Van Gieson's Picrofuchsin stain, showed that hybrids of CaHA‐CPM‐B, CaHA‐CPM‐V, and CaHA‐CPM‐I all resulted in collagen stimulation relative to baseline at 1 and 4 months, though the neocollagenesis was most significant in the CaHA‐CPM‐B hybrids (Figure 4B,C). The differences observed in these studies may be explained by the structure of the fillers: CaHA microspheres in the high strength CPM‐I and CPM‐V fillers are likely encapsulated in the HA gel, resulting in a gradual or linear biostimulatory response that correlates with the degradation of the HA component. Additionally, CaHA‐CPM‐B hybrids possess higher tan(*δ*) and thus likely spread more which is important for optimizing biostimulation [15, 31]. Relative to CaHA‐CPM hybrids, diluted CaHA‐CMC may facilitate more immediate interactions of CaHA microspheres, driving the initial exponential increase in dermal thickness seen in Bravo et al.'s studies. Consequently, one might expect hybrid CaHA‐CPM fillers to induce a more sustained, gradual biostimulatory effect due to the delayed release of CaHA microspheres from the carrier gel.

### 4.4 Limitations and Future Directions

This study is limited by its in vitro design, which may not fully reflect the behavior of CaHA‐CPM fillers in clinical settings, thus missing key features such as tissue integration and clinical duration of effect, which are not exclusively linked to gel rheological properties [32, 33]. Further, hybrid fillers have not been rigorously studied in vivo and lack regulatory approval. Additionally, only specific formulations of CPM‐HA and a single CaHA‐CMC product were evaluated, restricting the generalizability of the results to just CPM‐HA and CaHA‐CMC combinations. Future comparative research should include in vivo and clinical studies to validate the biostimulatory and clinical efficacy of these hybrid fillers. Expanding the range of HA and CaHA‐CMC products tested will help determine if the observed trends are consistent across different crosslinking technologies. Investigating the molecular interactions between CaHA microspheres and the HA matrix could also provide deeper insights into the mechanisms that enhance gel strength and longevity.

## 5 Conclusion

This study characterizes the rheological and physical properties of CaHA‐CPM hybrid fillers. The results indicate that both the type of CPM‐HA and the dilution ratio significantly influence key properties such as *G*′, *G*″, G*, tan *δ*, and η*, as well as cohesivity and extrusion force. The high stability and lack of CaHA sedimentation of these formulations may make them ideally suited for long‐term direct filling and gradual biostimulation. These properties are critical determinants of the fillers' performance in different tissue planes and their subsequent clinical applications and outcomes. By tailoring the composition of hybrid fillers, practitioners can optimize both immediate volumizing effects and long‐term regenerative benefits. Future in vivo and clinical studies are warranted to understand changes in biostimulatory mechanism of action, clinical duration of effect, and to establish standardized protocols for the clinical use of CaHA‐CPM hybrid fillers.

## Author Contributions

**Ewelina Kaczuba:** methodology, validation, formal analysis, investigation, resources, data curation, writing – review and editing. **Nabil Fakih‐Gomez:** validation, methodology, conceptualization, writing – original draft, writing – review and editing. **Jonathan Kadouch:** validation, conceptualization, writing – original draft, writing – review and editing. **Rolf Bartsch:** validation, conceptualization, writing – original draft, writing – review and editing. **Carla Pecora:** validation, conceptualization, writing – original draft, writing – review and editing. **Yana A. Yutskovskaya:** validation, conceptualization, data curation, resources, writing – original draft, writing – review and editing. **Nadine Hagedorn:** conceptualization, validation, formal analysis, resources, supervision, project administration, funding acquisition, writing – review and editing. **Sarah Backfisch:** methodology, validation, formal analysis, investigation, resources, data curation, writing – review and editing. **Alec D. McCarthy:** conceptualization, software, formal analysis, resources, data curation, writing – original draft, writing – review and editing, visualization, supervision, project administration. **Radia El‐Banna:** methodology, validation, formal analysis, investigation, resources, data curation, writing – review and editing.

## Conflicts of Interest

E.K., N.H., R.E.‐B., S.B. and A.D.M. are employed by Merz Aesthetics. N.F.‐G. is a consultant for Merz Aesthetics GmbH (Frankfurt, Germany). CdSP, Y.A.Y. and R.B. are speakers and consultants for Merz Aesthetics. J.K. is a speaker, trainer, and consultant for Merz Aesthetics and In Mode.

## Supporting information


**Table S1:** Weight compositions of different gels in this study.
**Table S2:** Pairwise comparisons of measured elastic moduli (*G*′).
**Table S3:** Pairwise comparisons of measured loss moduli (*G*″).
**Table S4:** Pairwise comparisons of measured viscosity.
**Table S5:** Pairwise comparisons of measured tan delta.
**Table S6:** Pairwise comparisons of measured complex moduli (G*).
**Table S7:** Pairwise comparisons of measured drop weight values.
**Table S8:** Pairwise comparisons of measured extrusion forces.
**Table S9:** Pairwise comparisons of measured axial strains.
**Table S10:** Pairwise comparisons of CaHA‐CPM‐V stability over a 24 h period.
**Table S11:** Pairwise comparisons of CaHA‐CPM‐I stability over a 24 h period.
**Table S12:** Pairwise comparisons of CaHA‐CPM‐B stability over a 24 h period.
**Table S13:** Pairwise comparisons of CaHA‐CPM‐R stability over a 24 h period.
**Table S14:** Pairwise comparisons of all hybrid stability immediately after preparation.
**Table S15:** Pairwise comparisons of all hybrid stability 24 h after preparation.
**Table S16:** Pairwise comparisons of biostimulatory data derived from Yutskovskaya et al. [7].

## Data Availability

The data that support the findings of this study are available from the corresponding author upon reasonable request.
