# Supplementary material for: Exploring the Rheology and Clinical Potential of Calcium Hydroxylapatite‐Hyaluronic Acid Hybrids
Source: J Cosmet Dermatol. 2025 Oct 13;24(10):e70473. doi: 10.1111/jocd.70473 (PMC12516938; doi:10.1111/jocd.70473)
Supplement: Supplementary file 1 — Table S1: Weight compositions of different gels in this study. Table S2: Pairwise comparisons of measured elastic moduli (G′). Table S3: Pairwise comparisons of measured loss moduli (G″). Table S4: Pairwise comparisons of measured viscosity. Table S5: Pairwise comparisons of measured tan delta. Table S6: Pairwise comparisons of measured complex moduli (G*). Table S7: Pairwise comparisons of measured drop weight values. Table S8: Pairwise comparisons of measured extrusion forces. Table S9: Pairwise comparisons of measured axial strains. Table S10: Pairwise comparisons of CaHA‐CPM‐V stability over a 24 h period. Table S11: Pairwise comparisons of CaHA‐CPM‐I stability over a 24 h period. Table S12: Pairwise comparisons of CaHA‐CPM‐B stability over a 24 h period. Table S13: Pairwise comparisons of CaHA‐CPM‐R stability over a 24 h period. Table S14: Pairwise comparisons of all hybrid stability immediately after preparation. Table S15: Pairwise comparisons of all hybrid stability 24 h after preparation. Table S16: Pairwise comparisons of biostimulatory data derived from Yutskovskaya et al. [7]. [file JOCD-24-e70473-s001.docx]

**Supporting Information**

**Exploring the Rheology and Clinical Potential of Calcium Hydroxylapatite-Hyaluronic Acid Hybrids**

**Table S1. Weight compositions of different gels in this study.**

| **Mixing ratio** | **CaHA in wt%** | **Gel carrier in wt%** |
| --- | --- | --- |
| **CaHA-CMC** | 57 | 43 |
| **CaHA-CMC + CPM-HA 1:1** | 41 | 59 |
| **CaHA-CMC + CPM-HA 1:2** | 32 | 68 |
| **CaHA-CMC + CPM-HA 1:3** | 26 | 74 |
| **CaHA-CMC + CPM-HA 1:4** | 22 | 78 |

**Table S2. Pairwise comparisons of measured elastic moduli (G’).**

| Tukey's multiple comparisons test | Mean Diff. | 95.00% CI of diff. | Below threshold? | Summary | Adjusted P Value |
| --- | --- | --- | --- | --- | --- |
|  |  |  |  |  |  |
| 1:1:CaHA-CPMB vs. 1:1:CaHA-CPMV | -352 | -387.6 to -316.4 | Yes | **** | <0.0001 |
| 1:1:CaHA-CPMB vs. 1:1:CaHA-CPMI | -207.6 | -243.3 to -172.0 | Yes | **** | <0.0001 |
| 1:1:CaHA-CPMB vs. 1:1:CaHA-CPMR | 90.65 | 55.05 to 126.3 | Yes | **** | <0.0001 |
| 1:1:CaHA-CPMB vs. 1:2:CaHA-CPMB | 172.3 | 136.7 to 207.9 | Yes | **** | <0.0001 |
| 1:1:CaHA-CPMB vs. 1:2:CaHA-CPMV | -144.6 | -180.2 to -109.0 | Yes | **** | <0.0001 |
| 1:1:CaHA-CPMB vs. 1:2:CaHA-CPMI | -56.06 | -91.67 to -20.45 | Yes | *** | 0.0002 |
| 1:1:CaHA-CPMB vs. 1:2:CaHA-CPMR | 211.4 | 175.8 to 247.0 | Yes | **** | <0.0001 |
| 1:1:CaHA-CPMB vs. 1:3:CaHA-CPMB | 236.9 | 201.3 to 272.5 | Yes | **** | <0.0001 |
| 1:1:CaHA-CPMB vs. 1:3:CaHA-CPMV | -72.88 | -108.5 to -37.28 | Yes | **** | <0.0001 |
| 1:1:CaHA-CPMB vs. 1:3:CaHA-CPMI | 11.97 | -23.64 to 47.58 | No | ns | 0.9954 |
| 1:1:CaHA-CPMB vs. 1:3:CaHA-CPMR | 248.2 | 212.6 to 283.8 | Yes | **** | <0.0001 |
| 1:1:CaHA-CPMB vs. 1:4:CaHA-CPMB | 251.4 | 215.8 to 287.0 | Yes | **** | <0.0001 |
| 1:1:CaHA-CPMB vs. 1:4:CaHA-CPMV | -37.72 | -73.32 to -2.110 | Yes | * | 0.0295 |
| 1:1:CaHA-CPMB vs. 1:4:CaHA-CPMI | 44.7 | 9.097 to 80.31 | Yes | ** | 0.0045 |
| 1:1:CaHA-CPMB vs. 1:4:CaHA-CPMR | 264.9 | 229.3 to 300.5 | Yes | **** | <0.0001 |
| 1:1:CaHA-CPMV vs. 1:1:CaHA-CPMI | 144.3 | 108.7 to 179.9 | Yes | **** | <0.0001 |
| 1:1:CaHA-CPMV vs. 1:1:CaHA-CPMR | 442.6 | 407.0 to 478.2 | Yes | **** | <0.0001 |
| 1:1:CaHA-CPMV vs. 1:2:CaHA-CPMB | 524.3 | 488.7 to 559.9 | Yes | **** | <0.0001 |
| 1:1:CaHA-CPMV vs. 1:2:CaHA-CPMV | 207.4 | 171.8 to 243.0 | Yes | **** | <0.0001 |
| 1:1:CaHA-CPMV vs. 1:2:CaHA-CPMI | 295.9 | 260.3 to 331.5 | Yes | **** | <0.0001 |
| 1:1:CaHA-CPMV vs. 1:2:CaHA-CPMR | 563.4 | 527.8 to 599.0 | Yes | **** | <0.0001 |
| 1:1:CaHA-CPMV vs. 1:3:CaHA-CPMB | 588.9 | 553.3 to 624.5 | Yes | **** | <0.0001 |
| 1:1:CaHA-CPMV vs. 1:3:CaHA-CPMV | 279.1 | 243.5 to 314.7 | Yes | **** | <0.0001 |
| 1:1:CaHA-CPMV vs. 1:3:CaHA-CPMI | 364 | 328.4 to 399.6 | Yes | **** | <0.0001 |
| 1:1:CaHA-CPMV vs. 1:3:CaHA-CPMR | 600.1 | 564.5 to 635.8 | Yes | **** | <0.0001 |
| 1:1:CaHA-CPMV vs. 1:4:CaHA-CPMB | 603.4 | 567.8 to 639.0 | Yes | **** | <0.0001 |
| 1:1:CaHA-CPMV vs. 1:4:CaHA-CPMV | 314.3 | 278.7 to 349.9 | Yes | **** | <0.0001 |
| 1:1:CaHA-CPMV vs. 1:4:CaHA-CPMI | 396.7 | 361.1 to 432.3 | Yes | **** | <0.0001 |
| 1:1:CaHA-CPMV vs. 1:4:CaHA-CPMR | 616.9 | 581.3 to 652.5 | Yes | **** | <0.0001 |
| 1:1:CaHA-CPMI vs. 1:1:CaHA-CPMR | 298.3 | 262.7 to 333.9 | Yes | **** | <0.0001 |
| 1:1:CaHA-CPMI vs. 1:2:CaHA-CPMB | 380 | 344.4 to 415.6 | Yes | **** | <0.0001 |
| 1:1:CaHA-CPMI vs. 1:2:CaHA-CPMV | 63.05 | 27.45 to 98.66 | Yes | **** | <0.0001 |
| 1:1:CaHA-CPMI vs. 1:2:CaHA-CPMI | 151.6 | 116.0 to 187.2 | Yes | **** | <0.0001 |
| 1:1:CaHA-CPMI vs. 1:2:CaHA-CPMR | 419.1 | 383.5 to 454.7 | Yes | **** | <0.0001 |
| 1:1:CaHA-CPMI vs. 1:3:CaHA-CPMB | 444.6 | 409.0 to 480.2 | Yes | **** | <0.0001 |
| 1:1:CaHA-CPMI vs. 1:3:CaHA-CPMV | 134.8 | 99.16 to 170.4 | Yes | **** | <0.0001 |
| 1:1:CaHA-CPMI vs. 1:3:CaHA-CPMI | 219.6 | 184.0 to 255.2 | Yes | **** | <0.0001 |
| 1:1:CaHA-CPMI vs. 1:3:CaHA-CPMR | 455.8 | 420.2 to 491.4 | Yes | **** | <0.0001 |
| 1:1:CaHA-CPMI vs. 1:4:CaHA-CPMB | 459.1 | 423.4 to 494.7 | Yes | **** | <0.0001 |
| 1:1:CaHA-CPMI vs. 1:4:CaHA-CPMV | 169.9 | 134.3 to 205.5 | Yes | **** | <0.0001 |
| 1:1:CaHA-CPMI vs. 1:4:CaHA-CPMI | 252.4 | 216.7 to 288.0 | Yes | **** | <0.0001 |
| 1:1:CaHA-CPMI vs. 1:4:CaHA-CPMR | 472.6 | 437.0 to 508.2 | Yes | **** | <0.0001 |
| 1:1:CaHA-CPMR vs. 1:2:CaHA-CPMB | 81.67 | 46.06 to 117.3 | Yes | **** | <0.0001 |
| 1:1:CaHA-CPMR vs. 1:2:CaHA-CPMV | -235.2 | -270.9 to -199.6 | Yes | **** | <0.0001 |
| 1:1:CaHA-CPMR vs. 1:2:CaHA-CPMI | -146.7 | -182.3 to -111.1 | Yes | **** | <0.0001 |
| 1:1:CaHA-CPMR vs. 1:2:CaHA-CPMR | 120.8 | 85.16 to 156.4 | Yes | **** | <0.0001 |
| 1:1:CaHA-CPMR vs. 1:3:CaHA-CPMB | 146.3 | 110.7 to 181.9 | Yes | **** | <0.0001 |
| 1:1:CaHA-CPMR vs. 1:3:CaHA-CPMV | -163.5 | -199.1 to -127.9 | Yes | **** | <0.0001 |
| 1:1:CaHA-CPMR vs. 1:3:CaHA-CPMI | -78.68 | -114.3 to -43.08 | Yes | **** | <0.0001 |
| 1:1:CaHA-CPMR vs. 1:3:CaHA-CPMR | 157.5 | 121.9 to 193.1 | Yes | **** | <0.0001 |
| 1:1:CaHA-CPMR vs. 1:4:CaHA-CPMB | 160.8 | 125.1 to 196.4 | Yes | **** | <0.0001 |
| 1:1:CaHA-CPMR vs. 1:4:CaHA-CPMV | -128.4 | -164.0 to -92.76 | Yes | **** | <0.0001 |
| 1:1:CaHA-CPMR vs. 1:4:CaHA-CPMI | -45.95 | -81.56 to -10.34 | Yes | ** | 0.0031 |
| 1:1:CaHA-CPMR vs. 1:4:CaHA-CPMR | 174.3 | 138.7 to 209.9 | Yes | **** | <0.0001 |
| 1:2:CaHA-CPMB vs. 1:2:CaHA-CPMV | -316.9 | -352.5 to -281.3 | Yes | **** | <0.0001 |
| 1:2:CaHA-CPMB vs. 1:2:CaHA-CPMI | -228.4 | -264.0 to -192.8 | Yes | **** | <0.0001 |
| 1:2:CaHA-CPMB vs. 1:2:CaHA-CPMR | 39.1 | 3.494 to 74.71 | Yes | * | 0.0206 |
| 1:2:CaHA-CPMB vs. 1:3:CaHA-CPMB | 64.6 | 28.99 to 100.2 | Yes | **** | <0.0001 |
| 1:2:CaHA-CPMB vs. 1:3:CaHA-CPMV | -245.2 | -280.8 to -209.6 | Yes | **** | <0.0001 |
| 1:2:CaHA-CPMB vs. 1:3:CaHA-CPMI | -160.4 | -196.0 to -124.7 | Yes | **** | <0.0001 |
| 1:2:CaHA-CPMB vs. 1:3:CaHA-CPMR | 75.83 | 40.23 to 111.4 | Yes | **** | <0.0001 |
| 1:2:CaHA-CPMB vs. 1:4:CaHA-CPMB | 79.08 | 43.48 to 114.7 | Yes | **** | <0.0001 |
| 1:2:CaHA-CPMB vs. 1:4:CaHA-CPMV | -210 | -245.6 to -174.4 | Yes | **** | <0.0001 |
| 1:2:CaHA-CPMB vs. 1:4:CaHA-CPMI | -127.6 | -163.2 to -92.01 | Yes | **** | <0.0001 |
| 1:2:CaHA-CPMB vs. 1:4:CaHA-CPMR | 92.6 | 57.00 to 128.2 | Yes | **** | <0.0001 |
| 1:2:CaHA-CPMV vs. 1:2:CaHA-CPMI | 88.53 | 52.93 to 124.1 | Yes | **** | <0.0001 |
| 1:2:CaHA-CPMV vs. 1:2:CaHA-CPMR | 356 | 320.4 to 391.6 | Yes | **** | <0.0001 |
| 1:2:CaHA-CPMV vs. 1:3:CaHA-CPMB | 381.5 | 345.9 to 417.1 | Yes | **** | <0.0001 |
| 1:2:CaHA-CPMV vs. 1:3:CaHA-CPMV | 71.71 | 36.10 to 107.3 | Yes | **** | <0.0001 |
| 1:2:CaHA-CPMV vs. 1:3:CaHA-CPMI | 156.6 | 121.0 to 192.2 | Yes | **** | <0.0001 |
| 1:2:CaHA-CPMV vs. 1:3:CaHA-CPMR | 392.7 | 357.1 to 428.4 | Yes | **** | <0.0001 |
| 1:2:CaHA-CPMV vs. 1:4:CaHA-CPMB | 396 | 360.4 to 431.6 | Yes | **** | <0.0001 |
| 1:2:CaHA-CPMV vs. 1:4:CaHA-CPMV | 106.9 | 71.27 to 142.5 | Yes | **** | <0.0001 |
| 1:2:CaHA-CPMV vs. 1:4:CaHA-CPMI | 189.3 | 153.7 to 224.9 | Yes | **** | <0.0001 |
| 1:2:CaHA-CPMV vs. 1:4:CaHA-CPMR | 409.5 | 373.9 to 445.1 | Yes | **** | <0.0001 |
| 1:2:CaHA-CPMI vs. 1:2:CaHA-CPMR | 267.5 | 231.9 to 303.1 | Yes | **** | <0.0001 |
| 1:2:CaHA-CPMI vs. 1:3:CaHA-CPMB | 293 | 257.4 to 328.6 | Yes | **** | <0.0001 |
| 1:2:CaHA-CPMI vs. 1:3:CaHA-CPMV | -16.82 | -52.43 to 18.78 | No | ns | 0.9157 |
| 1:2:CaHA-CPMI vs. 1:3:CaHA-CPMI | 68.03 | 32.42 to 103.6 | Yes | **** | <0.0001 |
| 1:2:CaHA-CPMI vs. 1:3:CaHA-CPMR | 304.2 | 268.6 to 339.8 | Yes | **** | <0.0001 |
| 1:2:CaHA-CPMI vs. 1:4:CaHA-CPMB | 307.5 | 271.9 to 343.1 | Yes | **** | <0.0001 |
| 1:2:CaHA-CPMI vs. 1:4:CaHA-CPMV | 18.34 | -17.26 to 53.95 | No | ns | 0.8527 |
| 1:2:CaHA-CPMI vs. 1:4:CaHA-CPMI | 100.8 | 65.16 to 136.4 | Yes | **** | <0.0001 |
| 1:2:CaHA-CPMI vs. 1:4:CaHA-CPMR | 321 | 285.4 to 356.6 | Yes | **** | <0.0001 |
| 1:2:CaHA-CPMR vs. 1:3:CaHA-CPMB | 25.5 | -10.11 to 61.11 | No | ns | 0.3986 |
| 1:2:CaHA-CPMR vs. 1:3:CaHA-CPMV | -284.3 | -319.9 to -248.7 | Yes | **** | <0.0001 |
| 1:2:CaHA-CPMR vs. 1:3:CaHA-CPMI | -199.5 | -235.1 to -163.8 | Yes | **** | <0.0001 |
| 1:2:CaHA-CPMR vs. 1:3:CaHA-CPMR | 36.73 | 1.127 to 72.34 | Yes | * | 0.0378 |
| 1:2:CaHA-CPMR vs. 1:4:CaHA-CPMB | 39.98 | 4.375 to 75.59 | Yes | * | 0.0163 |
| 1:2:CaHA-CPMR vs. 1:4:CaHA-CPMV | -249.1 | -284.7 to -213.5 | Yes | **** | <0.0001 |
| 1:2:CaHA-CPMR vs. 1:4:CaHA-CPMI | -166.7 | -202.3 to -131.1 | Yes | **** | <0.0001 |
| 1:2:CaHA-CPMR vs. 1:4:CaHA-CPMR | 53.5 | 17.90 to 89.11 | Yes | *** | 0.0004 |
| 1:3:CaHA-CPMB vs. 1:3:CaHA-CPMV | -309.8 | -345.4 to -274.2 | Yes | **** | <0.0001 |
| 1:3:CaHA-CPMB vs. 1:3:CaHA-CPMI | -225 | -260.6 to -189.3 | Yes | **** | <0.0001 |
| 1:3:CaHA-CPMB vs. 1:3:CaHA-CPMR | 11.23 | -24.37 to 46.84 | No | ns | 0.9976 |
| 1:3:CaHA-CPMB vs. 1:4:CaHA-CPMB | 14.48 | -21.12 to 50.09 | No | ns | 0.9732 |
| 1:3:CaHA-CPMB vs. 1:4:CaHA-CPMV | -274.6 | -310.2 to -239.0 | Yes | **** | <0.0001 |
| 1:3:CaHA-CPMB vs. 1:4:CaHA-CPMI | -192.2 | -227.8 to -156.6 | Yes | **** | <0.0001 |
| 1:3:CaHA-CPMB vs. 1:4:CaHA-CPMR | 28 | -7.605 to 63.61 | No | ns | 0.2609 |
| 1:3:CaHA-CPMV vs. 1:3:CaHA-CPMI | 84.85 | 49.25 to 120.5 | Yes | **** | <0.0001 |
| 1:3:CaHA-CPMV vs. 1:3:CaHA-CPMR | 321 | 285.4 to 356.6 | Yes | **** | <0.0001 |
| 1:3:CaHA-CPMV vs. 1:4:CaHA-CPMB | 324.3 | 288.7 to 359.9 | Yes | **** | <0.0001 |
| 1:3:CaHA-CPMV vs. 1:4:CaHA-CPMV | 35.17 | -0.4396 to 70.77 | No | ns | 0.0557 |
| 1:3:CaHA-CPMV vs. 1:4:CaHA-CPMI | 117.6 | 81.98 to 153.2 | Yes | **** | <0.0001 |
| 1:3:CaHA-CPMV vs. 1:4:CaHA-CPMR | 337.8 | 302.2 to 373.4 | Yes | **** | <0.0001 |
| 1:3:CaHA-CPMI vs. 1:3:CaHA-CPMR | 236.2 | 200.6 to 271.8 | Yes | **** | <0.0001 |
| 1:3:CaHA-CPMI vs. 1:4:CaHA-CPMB | 239.4 | 203.8 to 275.0 | Yes | **** | <0.0001 |
| 1:3:CaHA-CPMI vs. 1:4:CaHA-CPMV | -49.69 | -85.29 to -14.08 | Yes | ** | 0.0011 |
| 1:3:CaHA-CPMI vs. 1:4:CaHA-CPMI | 32.73 | -2.873 to 68.34 | No | ns | 0.0985 |
| 1:3:CaHA-CPMI vs. 1:4:CaHA-CPMR | 253 | 217.3 to 288.6 | Yes | **** | <0.0001 |
| 1:3:CaHA-CPMR vs. 1:4:CaHA-CPMB | 3.249 | -32.36 to 38.85 | No | ns | >0.9999 |
| 1:3:CaHA-CPMR vs. 1:4:CaHA-CPMV | -285.9 | -321.5 to -250.3 | Yes | **** | <0.0001 |
| 1:3:CaHA-CPMR vs. 1:4:CaHA-CPMI | -203.5 | -239.1 to -167.8 | Yes | **** | <0.0001 |
| 1:3:CaHA-CPMR vs. 1:4:CaHA-CPMR | 16.77 | -18.84 to 52.37 | No | ns | 0.9176 |
| 1:4:CaHA-CPMB vs. 1:4:CaHA-CPMV | -289.1 | -324.7 to -253.5 | Yes | **** | <0.0001 |
| 1:4:CaHA-CPMB vs. 1:4:CaHA-CPMI | -206.7 | -242.3 to -171.1 | Yes | **** | <0.0001 |
| 1:4:CaHA-CPMB vs. 1:4:CaHA-CPMR | 13.52 | -22.09 to 49.13 | No | ns | 0.9853 |
| 1:4:CaHA-CPMV vs. 1:4:CaHA-CPMI | 82.42 | 46.81 to 118.0 | Yes | **** | <0.0001 |
| 1:4:CaHA-CPMV vs. 1:4:CaHA-CPMR | 302.6 | 267.0 to 338.2 | Yes | **** | <0.0001 |
| 1:4:CaHA-CPMI vs. 1:4:CaHA-CPMR | 220.2 | 184.6 to 255.8 | Yes | **** | <0.0001 |

**Table S3. Pairwise comparisons of measured loss moduli (G”).**

|  |  |  |  |  |  |
| --- | --- | --- | --- | --- | --- |
|  |  |  |  |  |  |
| Tukey's multiple comparisons test | Mean Diff. | 95.00% CI of diff. | Below threshold? | Summary | Adjusted P Value |
|  |  |  |  |  |  |
| CaHA-CMC Only | |  |  |  |  |
| CaHA-CPMB vs. CaHA-CPMV | 0 | -19.95 to 19.95 | No | ns | >0.9999 |
| CaHA-CPMB vs. CaHA-CPMI | 0 | -19.95 to 19.95 | No | ns | >0.9999 |
| CaHA-CPMB vs. CaHA-CPMR | 0 | -19.95 to 19.95 | No | ns | >0.9999 |
| CaHA-CPMV vs. CaHA-CPMI | 0 | -19.95 to 19.95 | No | ns | >0.9999 |
| CaHA-CPMV vs. CaHA-CPMR | 0 | -19.95 to 19.95 | No | ns | >0.9999 |
| CaHA-CPMI vs. CaHA-CPMR | 0 | -19.95 to 19.95 | No | ns | >0.9999 |
|  |  |  |  |  |  |
| 1:1 |  |  |  |  |  |
| CaHA-CPMB vs. CaHA-CPMV | -97.08 | -117.0 to -77.13 | Yes | **** | <0.0001 |
| CaHA-CPMB vs. CaHA-CPMI | -45.89 | -65.84 to -25.94 | Yes | **** | <0.0001 |
| CaHA-CPMB vs. CaHA-CPMR | 26.34 | 6.387 to 46.29 | Yes | ** | 0.0052 |
| CaHA-CPMV vs. CaHA-CPMI | 51.19 | 31.24 to 71.15 | Yes | **** | <0.0001 |
| CaHA-CPMV vs. CaHA-CPMR | 123.4 | 103.5 to 143.4 | Yes | **** | <0.0001 |
| CaHA-CPMI vs. CaHA-CPMR | 72.23 | 52.28 to 92.18 | Yes | **** | <0.0001 |
|  |  |  |  |  |  |
| 1:2 |  |  |  |  |  |
| CaHA-CPMB vs. CaHA-CPMV | -70.28 | -90.23 to -50.33 | Yes | **** | <0.0001 |
| CaHA-CPMB vs. CaHA-CPMI | -57.8 | -77.76 to -37.85 | Yes | **** | <0.0001 |
| CaHA-CPMB vs. CaHA-CPMR | 7.954 | -12.00 to 27.91 | No | ns | 0.7146 |
| CaHA-CPMV vs. CaHA-CPMI | 12.47 | -7.479 to 32.43 | No | ns | 0.3537 |
| CaHA-CPMV vs. CaHA-CPMR | 78.23 | 58.28 to 98.19 | Yes | **** | <0.0001 |
| CaHA-CPMI vs. CaHA-CPMR | 65.76 | 45.81 to 85.71 | Yes | **** | <0.0001 |
|  |  |  |  |  |  |
| 1:3 |  |  |  |  |  |
| CaHA-CPMB vs. CaHA-CPMV | -65.84 | -85.79 to -45.89 | Yes | **** | <0.0001 |
| CaHA-CPMB vs. CaHA-CPMI | -55.57 | -75.52 to -35.62 | Yes | **** | <0.0001 |
| CaHA-CPMB vs. CaHA-CPMR | -1.12 | -21.07 to 18.83 | No | ns | 0.9988 |
| CaHA-CPMV vs. CaHA-CPMI | 10.27 | -9.682 to 30.22 | No | ns | 0.524 |
| CaHA-CPMV vs. CaHA-CPMR | 64.72 | 44.77 to 84.67 | Yes | **** | <0.0001 |
| CaHA-CPMI vs. CaHA-CPMR | 54.45 | 34.50 to 74.40 | Yes | **** | <0.0001 |
|  |  |  |  |  |  |
| 1:4 |  |  |  |  |  |
| CaHA-CPMB vs. CaHA-CPMV | -55 | -74.95 to -35.05 | Yes | **** | <0.0001 |
| CaHA-CPMB vs. CaHA-CPMI | -46.52 | -66.47 to -26.57 | Yes | **** | <0.0001 |
| CaHA-CPMB vs. CaHA-CPMR | -2.535 | -22.49 to 17.42 | No | ns | 0.9865 |
| CaHA-CPMV vs. CaHA-CPMI | 8.48 | -11.47 to 28.43 | No | ns | 0.6723 |
| CaHA-CPMV vs. CaHA-CPMR | 52.46 | 32.51 to 72.42 | Yes | **** | <0.0001 |
| CaHA-CPMI vs. CaHA-CPMR | 43.98 | 24.03 to 63.94 | Yes | **** | <0.0001 |
|  |  |  |  |  |  |
| CPM Only | |  |  |  |  |
| CaHA-CPMB vs. CaHA-CPMV | -26.54 | -46.49 to -6.589 | Yes | ** | 0.0048 |
| CaHA-CPMB vs. CaHA-CPMI | -29.34 | -49.30 to -9.391 | Yes | ** | 0.0016 |
| CaHA-CPMB vs. CaHA-CPMR | 13.93 | -6.021 to 33.88 | No | ns | 0.2595 |
| CaHA-CPMV vs. CaHA-CPMI | -2.802 | -22.75 to 17.15 | No | ns | 0.982 |
| CaHA-CPMV vs. CaHA-CPMR | 40.47 | 20.52 to 60.43 | Yes | **** | <0.0001 |
| CaHA-CPMI vs. CaHA-CPMR | 43.28 | 23.32 to 63.23 | Yes | **** | <0.0001 |
|  |  |  |  |  |  |
| CaHA-CPMB | |  |  |  |  |
| CaHA-CMC Only vs. 1:1 | 671.7 | 649.5 to 694.0 | Yes | **** | <0.0001 |
| CaHA-CMC Only vs. 1:2 | 758.7 | 736.5 to 781.0 | Yes | **** | <0.0001 |
| CaHA-CMC Only vs. 1:3 | 790.7 | 768.4 to 812.9 | Yes | **** | <0.0001 |
| CaHA-CMC Only vs. 1:4 | 800.4 | 778.2 to 822.7 | Yes | **** | <0.0001 |
| CaHA-CMC Only vs. CPM Only | 837.8 | 815.6 to 860.1 | Yes | **** | <0.0001 |
| 1:1 vs. 1:2 | 87.01 | 64.76 to 109.3 | Yes | **** | <0.0001 |
| 1:1 vs. 1:3 | 118.9 | 96.70 to 141.2 | Yes | **** | <0.0001 |
| 1:1 vs. 1:4 | 128.7 | 106.5 to 151.0 | Yes | **** | <0.0001 |
| 1:1 vs. CPM Only | 166.1 | 143.8 to 188.3 | Yes | **** | <0.0001 |
| 1:2 vs. 1:3 | 31.94 | 9.686 to 54.19 | Yes | ** | 0.0013 |
| 1:2 vs. 1:4 | 41.69 | 19.44 to 63.94 | Yes | **** | <0.0001 |
| 1:2 vs. CPM Only | 79.08 | 56.83 to 101.3 | Yes | **** | <0.0001 |
| 1:3 vs. 1:4 | 9.757 | -12.49 to 32.01 | No | ns | 0.7828 |
| 1:3 vs. CPM Only | 47.14 | 24.89 to 69.39 | Yes | **** | <0.0001 |
| 1:4 vs. CPM Only | 37.38 | 15.13 to 59.64 | Yes | *** | 0.0001 |
|  |  |  |  |  |  |
| CaHA-CPMV | |  |  |  |  |
| CaHA-CMC Only vs. 1:1 | 574.7 | 552.4 to 596.9 | Yes | **** | <0.0001 |
| CaHA-CMC Only vs. 1:2 | 688.5 | 666.2 to 710.7 | Yes | **** | <0.0001 |
| CaHA-CMC Only vs. 1:3 | 724.8 | 702.6 to 747.1 | Yes | **** | <0.0001 |
| CaHA-CMC Only vs. 1:4 | 745.4 | 723.2 to 767.7 | Yes | **** | <0.0001 |
| CaHA-CMC Only vs. CPM Only | 811.3 | 789.0 to 833.5 | Yes | **** | <0.0001 |
| 1:1 vs. 1:2 | 113.8 | 91.56 to 136.1 | Yes | **** | <0.0001 |
| 1:1 vs. 1:3 | 150.2 | 127.9 to 172.4 | Yes | **** | <0.0001 |
| 1:1 vs. 1:4 | 170.8 | 148.5 to 193.0 | Yes | **** | <0.0001 |
| 1:1 vs. CPM Only | 236.6 | 214.4 to 258.9 | Yes | **** | <0.0001 |
| 1:2 vs. 1:3 | 36.37 | 14.12 to 58.62 | Yes | *** | 0.0002 |
| 1:2 vs. 1:4 | 56.97 | 34.72 to 79.22 | Yes | **** | <0.0001 |
| 1:2 vs. CPM Only | 122.8 | 100.6 to 145.1 | Yes | **** | <0.0001 |
| 1:3 vs. 1:4 | 20.6 | -1.651 to 42.85 | No | ns | 0.0843 |
| 1:3 vs. CPM Only | 86.44 | 64.19 to 108.7 | Yes | **** | <0.0001 |
| 1:4 vs. CPM Only | 65.84 | 43.59 to 88.09 | Yes | **** | <0.0001 |
|  |  |  |  |  |  |
| CaHA-CPMI | |  |  |  |  |
| CaHA-CMC Only vs. 1:1 | 625.8 | 603.6 to 648.1 | Yes | **** | <0.0001 |
| CaHA-CMC Only vs. 1:2 | 700.9 | 678.7 to 723.2 | Yes | **** | <0.0001 |
| CaHA-CMC Only vs. 1:3 | 735.1 | 712.9 to 757.4 | Yes | **** | <0.0001 |
| CaHA-CMC Only vs. 1:4 | 753.9 | 731.7 to 776.2 | Yes | **** | <0.0001 |
| CaHA-CMC Only vs. CPM Only | 808.5 | 786.2 to 830.7 | Yes | **** | <0.0001 |
| 1:1 vs. 1:2 | 75.1 | 52.84 to 97.35 | Yes | **** | <0.0001 |
| 1:1 vs. 1:3 | 109.3 | 87.02 to 131.5 | Yes | **** | <0.0001 |
| 1:1 vs. 1:4 | 128.1 | 105.8 to 150.3 | Yes | **** | <0.0001 |
| 1:1 vs. CPM Only | 182.6 | 160.4 to 204.9 | Yes | **** | <0.0001 |
| 1:2 vs. 1:3 | 34.17 | 11.92 to 56.42 | Yes | *** | 0.0005 |
| 1:2 vs. 1:4 | 52.98 | 30.73 to 75.23 | Yes | **** | <0.0001 |
| 1:2 vs. CPM Only | 107.5 | 85.29 to 129.8 | Yes | **** | <0.0001 |
| 1:3 vs. 1:4 | 18.81 | -3.441 to 41.06 | No | ns | 0.1419 |
| 1:3 vs. CPM Only | 73.37 | 51.12 to 95.62 | Yes | **** | <0.0001 |
| 1:4 vs. CPM Only | 54.56 | 32.31 to 76.81 | Yes | **** | <0.0001 |
|  |  |  |  |  |  |
| CaHA-CPMR | |  |  |  |  |
| CaHA-CMC Only vs. 1:1 | 698.1 | 675.8 to 720.3 | Yes | **** | <0.0001 |
| CaHA-CMC Only vs. 1:2 | 766.7 | 744.5 to 789.0 | Yes | **** | <0.0001 |
| CaHA-CMC Only vs. 1:3 | 789.6 | 767.3 to 811.8 | Yes | **** | <0.0001 |
| CaHA-CMC Only vs. 1:4 | 797.9 | 775.7 to 820.2 | Yes | **** | <0.0001 |
| CaHA-CMC Only vs. CPM Only | 851.8 | 829.5 to 874.0 | Yes | **** | <0.0001 |
| 1:1 vs. 1:2 | 68.62 | 46.37 to 90.88 | Yes | **** | <0.0001 |
| 1:1 vs. 1:3 | 91.49 | 69.24 to 113.7 | Yes | **** | <0.0001 |
| 1:1 vs. 1:4 | 99.83 | 77.58 to 122.1 | Yes | **** | <0.0001 |
| 1:1 vs. CPM Only | 153.7 | 131.4 to 175.9 | Yes | **** | <0.0001 |
| 1:2 vs. 1:3 | 22.86 | 0.6114 to 45.11 | Yes | * | 0.0408 |
| 1:2 vs. 1:4 | 31.2 | 8.954 to 53.46 | Yes | ** | 0.0017 |
| 1:2 vs. CPM Only | 85.06 | 62.80 to 107.3 | Yes | **** | <0.0001 |
| 1:3 vs. 1:4 | 8.342 | -13.91 to 30.59 | No | ns | 0.8737 |
| 1:3 vs. CPM Only | 62.19 | 39.94 to 84.44 | Yes | **** | <0.0001 |
| 1:4 vs. CPM Only | 53.85 | 31.60 to 76.10 | Yes | **** | <0.0001 |

**Table S4. Pairwise comparisons of measured viscosity.**

|  |  |  |  |  |  |
| --- | --- | --- | --- | --- | --- |
|  |  |  |  |  |  |
| **Tukey's multiple comparisons test** | **Mean Diff.** | **95.00% CI of diff.** | **Below threshold?** | **Summary** | **Adjusted P Value** |
|  |  |  |  |  |  |
| **CaHA-CMC Only** | |  |  |  |  |
| **CaHA-CPMB vs. CaHA-CPMV** | **0** | **-9.453 to 9.453** | **No** | **ns** | **>0.9999** |
| **CaHA-CPMB vs. CaHA-CPMI** | **0** | **-9.453 to 9.453** | **No** | **ns** | **>0.9999** |
| **CaHA-CPMB vs. CaHA-CPMR** | **0** | **-9.453 to 9.453** | **No** | **ns** | **>0.9999** |
| **CaHA-CPMV vs. CaHA-CPMI** | **0** | **-9.453 to 9.453** | **No** | **ns** | **>0.9999** |
| **CaHA-CPMV vs. CaHA-CPMR** | **0** | **-9.453 to 9.453** | **No** | **ns** | **>0.9999** |
| **CaHA-CPMI vs. CaHA-CPMR** | **0** | **-9.453 to 9.453** | **No** | **ns** | **>0.9999** |
|  |  |  |  |  |  |
| **1:1** |  |  |  |  |  |
| **CaHA-CPMB vs. CaHA-CPMV** | **-57.17** | **-66.62 to -47.71** | **Yes** | ******** | **<0.0001** |
| **CaHA-CPMB vs. CaHA-CPMI** | **-32.86** | **-42.31 to -23.40** | **Yes** | ******** | **<0.0001** |
| **CaHA-CPMB vs. CaHA-CPMR** | **14.49** | **5.037 to 23.94** | **Yes** | ******* | **0.0009** |
| **CaHA-CPMV vs. CaHA-CPMI** | **24.31** | **14.86 to 33.76** | **Yes** | ******** | **<0.0001** |
| **CaHA-CPMV vs. CaHA-CPMR** | **71.66** | **62.20 to 81.11** | **Yes** | ******** | **<0.0001** |
| **CaHA-CPMI vs. CaHA-CPMR** | **47.35** | **37.89 to 56.80** | **Yes** | ******** | **<0.0001** |
|  |  |  |  |  |  |
| **1:2** |  |  |  |  |  |
| **CaHA-CPMB vs. CaHA-CPMV** | **-50.45** | **-59.90 to -41.00** | **Yes** | ******** | **<0.0001** |
| **CaHA-CPMB vs. CaHA-CPMI** | **-36.61** | **-46.06 to -27.16** | **Yes** | ******** | **<0.0001** |
| **CaHA-CPMB vs. CaHA-CPMR** | **5.853** | **-3.599 to 15.31** | **No** | **ns** | **0.3622** |
| **CaHA-CPMV vs. CaHA-CPMI** | **13.84** | **4.387 to 23.29** | **Yes** | ****** | **0.0017** |
| **CaHA-CPMV vs. CaHA-CPMR** | **56.3** | **46.85 to 65.76** | **Yes** | ******** | **<0.0001** |
| **CaHA-CPMI vs. CaHA-CPMR** | **42.46** | **33.01 to 51.92** | **Yes** | ******** | **<0.0001** |
|  |  |  |  |  |  |
| **1:3** |  |  |  |  |  |
| **CaHA-CPMB vs. CaHA-CPMV** | **-49.13** | **-58.59 to -39.68** | **Yes** | ******** | **<0.0001** |
| **CaHA-CPMB vs. CaHA-CPMI** | **-35.91** | **-45.37 to -26.46** | **Yes** | ******** | **<0.0001** |
| **CaHA-CPMB vs. CaHA-CPMR** | **1.32** | **-8.133 to 10.77** | **No** | **ns** | **0.9823** |
| **CaHA-CPMV vs. CaHA-CPMI** | **13.22** | **3.767 to 22.67** | **Yes** | ****** | **0.0028** |
| **CaHA-CPMV vs. CaHA-CPMR** | **50.45** | **41.00 to 59.91** | **Yes** | ******** | **<0.0001** |
| **CaHA-CPMI vs. CaHA-CPMR** | **37.23** | **27.78 to 46.69** | **Yes** | ******** | **<0.0001** |
|  |  |  |  |  |  |
| **1:4** |  |  |  |  |  |
| **CaHA-CPMB vs. CaHA-CPMV** | **-45.57** | **-55.02 to -36.11** | **Yes** | ******** | **<0.0001** |
| **CaHA-CPMB vs. CaHA-CPMI** | **-32.74** | **-42.19 to -23.29** | **Yes** | ******** | **<0.0001** |
| **CaHA-CPMB vs. CaHA-CPMR** | **1.453** | **-7.999 to 10.91** | **No** | **ns** | **0.9766** |
| **CaHA-CPMV vs. CaHA-CPMI** | **12.83** | **3.374 to 22.28** | **Yes** | ****** | **0.0039** |
| **CaHA-CPMV vs. CaHA-CPMR** | **47.02** | **37.57 to 56.47** | **Yes** | ******** | **<0.0001** |
| **CaHA-CPMI vs. CaHA-CPMR** | **34.19** | **24.74 to 43.65** | **Yes** | ******** | **<0.0001** |
|  |  |  |  |  |  |
| **CPM Only** | |  |  |  |  |
| **CaHA-CPMB vs. CaHA-CPMV** | **-33.22** | **-42.67 to -23.76** | **Yes** | ******** | **<0.0001** |
| **CaHA-CPMB vs. CaHA-CPMI** | **-22.33** | **-31.78 to -12.88** | **Yes** | ******** | **<0.0001** |
| **CaHA-CPMB vs. CaHA-CPMR** | **4.24** | **-5.213 to 13.69** | **No** | **ns** | **0.6337** |
| **CaHA-CPMV vs. CaHA-CPMI** | **10.89** | **1.433 to 20.34** | **Yes** | ***** | **0.0181** |
| **CaHA-CPMV vs. CaHA-CPMR** | **37.46** | **28.00 to 46.91** | **Yes** | ******** | **<0.0001** |
| **CaHA-CPMI vs. CaHA-CPMR** | **26.57** | **17.12 to 36.02** | **Yes** | ******** | **<0.0001** |
|  |  |  |  |  |  |
| **CaHA-CPMB** | |  |  |  |  |
| **CaHA-CMC Only vs. 1:1** | **321.5** | **311.0 to 332.1** | **Yes** | ******** | **<0.0001** |
| **CaHA-CMC Only vs. 1:2** | **352.2** | **341.6 to 362.7** | **Yes** | ******** | **<0.0001** |
| **CaHA-CMC Only vs. 1:3** | **363.5** | **353.0 to 374.1** | **Yes** | ******** | **<0.0001** |
| **CaHA-CMC Only vs. 1:4** | **366.3** | **355.8 to 376.9** | **Yes** | ******** | **<0.0001** |
| **CaHA-CMC Only vs. CPM Only** | **377.3** | **366.8 to 387.9** | **Yes** | ******** | **<0.0001** |
| **1:1 vs. 1:2** | **30.65** | **20.11 to 41.19** | **Yes** | ******** | **<0.0001** |
| **1:1 vs. 1:3** | **42.03** | **31.49 to 52.57** | **Yes** | ******** | **<0.0001** |
| **1:1 vs. 1:4** | **44.8** | **34.26 to 55.34** | **Yes** | ******** | **<0.0001** |
| **1:1 vs. CPM Only** | **55.8** | **45.26 to 66.34** | **Yes** | ******** | **<0.0001** |
| **1:2 vs. 1:3** | **11.38** | **0.8386 to 21.92** | **Yes** | ***** | **0.0274** |
| **1:2 vs. 1:4** | **14.15** | **3.612 to 24.69** | **Yes** | ****** | **0.003** |
| **1:2 vs. CPM Only** | **25.15** | **14.61 to 35.69** | **Yes** | ******** | **<0.0001** |
| **1:3 vs. 1:4** | **2.773** | **-7.768 to 13.31** | **No** | **ns** | **0.9695** |
| **1:3 vs. CPM Only** | **13.77** | **3.227 to 24.31** | **Yes** | ****** | **0.0041** |
| **1:4 vs. CPM Only** | **11** | **0.4539 to 21.54** | **Yes** | ***** | **0.0363** |
|  |  |  |  |  |  |
| **CaHA-CPMV** | |  |  |  |  |
| **CaHA-CMC Only vs. 1:1** | **264.3** | **253.8 to 274.9** | **Yes** | ******** | **<0.0001** |
| **CaHA-CMC Only vs. 1:2** | **301.7** | **291.2 to 312.3** | **Yes** | ******** | **<0.0001** |
| **CaHA-CMC Only vs. 1:3** | **314.4** | **303.9 to 324.9** | **Yes** | ******** | **<0.0001** |
| **CaHA-CMC Only vs. 1:4** | **320.7** | **310.2 to 331.3** | **Yes** | ******** | **<0.0001** |
| **CaHA-CMC Only vs. CPM Only** | **344.1** | **333.6 to 354.6** | **Yes** | ******** | **<0.0001** |
| **1:1 vs. 1:2** | **37.37** | **26.83 to 47.91** | **Yes** | ******** | **<0.0001** |
| **1:1 vs. 1:3** | **50.06** | **39.52 to 60.60** | **Yes** | ******** | **<0.0001** |
| **1:1 vs. 1:4** | **56.4** | **45.86 to 66.94** | **Yes** | ******** | **<0.0001** |
| **1:1 vs. CPM Only** | **79.75** | **69.21 to 90.29** | **Yes** | ******** | **<0.0001** |
| **1:2 vs. 1:3** | **12.7** | **2.155 to 23.24** | **Yes** | ****** | **0.0099** |
| **1:2 vs. 1:4** | **19.04** | **8.495 to 29.58** | **Yes** | ******** | **<0.0001** |
| **1:2 vs. CPM Only** | **42.38** | **31.84 to 52.92** | **Yes** | ******** | **<0.0001** |
| **1:3 vs. 1:4** | **6.34** | **-4.201 to 16.88** | **No** | **ns** | **0.4848** |
| **1:3 vs. CPM Only** | **29.69** | **19.14 to 40.23** | **Yes** | ******** | **<0.0001** |
| **1:4 vs. CPM Only** | **23.35** | **12.80 to 33.89** | **Yes** | ******** | **<0.0001** |
|  |  |  |  |  |  |
| **CaHA-CPMI** | |  |  |  |  |
| **CaHA-CMC Only vs. 1:1** | **288.7** | **278.1 to 299.2** | **Yes** | ******** | **<0.0001** |
| **CaHA-CMC Only vs. 1:2** | **315.6** | **305.0 to 326.1** | **Yes** | ******** | **<0.0001** |
| **CaHA-CMC Only vs. 1:3** | **327.6** | **317.1 to 338.2** | **Yes** | ******** | **<0.0001** |
| **CaHA-CMC Only vs. 1:4** | **333.6** | **323.0 to 344.1** | **Yes** | ******** | **<0.0001** |
| **CaHA-CMC Only vs. CPM Only** | **355** | **344.4 to 365.5** | **Yes** | ******** | **<0.0001** |
| **1:1 vs. 1:2** | **26.9** | **16.36 to 37.44** | **Yes** | ******** | **<0.0001** |
| **1:1 vs. 1:3** | **38.97** | **28.43 to 49.51** | **Yes** | ******** | **<0.0001** |
| **1:1 vs. 1:4** | **44.92** | **34.38 to 55.46** | **Yes** | ******** | **<0.0001** |
| **1:1 vs. CPM Only** | **66.33** | **55.78 to 76.87** | **Yes** | ******** | **<0.0001** |
| **1:2 vs. 1:3** | **12.08** | **1.535 to 22.62** | **Yes** | ***** | **0.0162** |
| **1:2 vs. 1:4** | **18.02** | **7.482 to 28.56** | **Yes** | ******** | **<0.0001** |
| **1:2 vs. CPM Only** | **39.43** | **28.89 to 49.97** | **Yes** | ******** | **<0.0001** |
| **1:3 vs. 1:4** | **5.947** | **-4.595 to 16.49** | **No** | **ns** | **0.555** |
| **1:3 vs. CPM Only** | **27.35** | **16.81 to 37.89** | **Yes** | ******** | **<0.0001** |
| **1:4 vs. CPM Only** | **21.41** | **10.86 to 31.95** | **Yes** | ******** | **<0.0001** |
|  |  |  |  |  |  |
| **CaHA-CPMR** | |  |  |  |  |
| **CaHA-CMC Only vs. 1:1** | **336** | **325.5 to 346.5** | **Yes** | ******** | **<0.0001** |
| **CaHA-CMC Only vs. 1:2** | **358** | **347.5 to 368.6** | **Yes** | ******** | **<0.0001** |
| **CaHA-CMC Only vs. 1:3** | **364.9** | **354.3 to 375.4** | **Yes** | ******** | **<0.0001** |
| **CaHA-CMC Only vs. 1:4** | **367.8** | **357.2 to 378.3** | **Yes** | ******** | **<0.0001** |
| **CaHA-CMC Only vs. CPM Only** | **381.5** | **371.0 to 392.1** | **Yes** | ******** | **<0.0001** |
| **1:1 vs. 1:2** | **22.01** | **11.47 to 32.55** | **Yes** | ******** | **<0.0001** |
| **1:1 vs. 1:3** | **28.86** | **18.32 to 39.40** | **Yes** | ******** | **<0.0001** |
| **1:1 vs. 1:4** | **31.77** | **21.23 to 42.31** | **Yes** | ******** | **<0.0001** |
| **1:1 vs. CPM Only** | **45.55** | **35.01 to 56.09** | **Yes** | ******** | **<0.0001** |
| **1:2 vs. 1:3** | **6.847** | **-3.695 to 17.39** | **No** | **ns** | **0.3984** |
| **1:2 vs. 1:4** | **9.753** | **-0.7881 to 20.29** | **No** | **ns** | **0.0846** |
| **1:2 vs. CPM Only** | **23.54** | **12.99 to 34.08** | **Yes** | ******** | **<0.0001** |
| **1:3 vs. 1:4** | **2.907** | **-7.635 to 13.45** | **No** | **ns** | **0.9627** |
| **1:3 vs. CPM Only** | **16.69** | **6.147 to 27.23** | **Yes** | ******* | **0.0003** |
| **1:4 vs. CPM Only** | **13.78** | **3.241 to 24.32** | **Yes** | ****** | **0.0041** |

**Table S5. Pairwise comparisons of measured tan delta.**

| **Tukey's multiple comparisons test** | **Mean Diff.** | **95.00% CI of diff.** | **Below threshold?** | **Summary** | **Adjusted P Value** |
| --- | --- | --- | --- | --- | --- |
|  |  |  |  |  |  |
| **CaHA-CMC Only** | |  |  |  |  |
| **CaHA-CPMB vs. CaHA-CPMV** | **0** | **-0.03514 to 0.03514** | **No** | **ns** | **>0.9999** |
| **CaHA-CPMB vs. CaHA-CPMI** | **0** | **-0.03514 to 0.03514** | **No** | **ns** | **>0.9999** |
| **CaHA-CPMB vs. CaHA-CPMR** | **0** | **-0.03514 to 0.03514** | **No** | **ns** | **>0.9999** |
| **CaHA-CPMV vs. CaHA-CPMI** | **0** | **-0.03514 to 0.03514** | **No** | **ns** | **>0.9999** |
| **CaHA-CPMV vs. CaHA-CPMR** | **0** | **-0.03514 to 0.03514** | **No** | **ns** | **>0.9999** |
| **CaHA-CPMI vs. CaHA-CPMR** | **0** | **-0.03514 to 0.03514** | **No** | **ns** | **>0.9999** |
|  |  |  |  |  |  |
| **1:1** |  |  |  |  |  |
| **CaHA-CPMB vs. CaHA-CPMV** | **0.1467** | **0.1115 to 0.1818** | **Yes** | ******** | **<0.0001** |
| **CaHA-CPMB vs. CaHA-CPMI** | **0.1293** | **0.09420 to 0.1645** | **Yes** | ******** | **<0.0001** |
| **CaHA-CPMB vs. CaHA-CPMR** | **-0.09767** | **-0.1328 to -0.06253** | **Yes** | ******** | **<0.0001** |
| **CaHA-CPMV vs. CaHA-CPMI** | **-0.01733** | **-0.05247 to 0.01780** | **No** | **ns** | **0.5594** |
| **CaHA-CPMV vs. CaHA-CPMR** | **-0.2443** | **-0.2795 to -0.2092** | **Yes** | ******** | **<0.0001** |
| **CaHA-CPMI vs. CaHA-CPMR** | **-0.227** | **-0.2621 to -0.1919** | **Yes** | ******** | **<0.0001** |
|  |  |  |  |  |  |
| **1:2** |  |  |  |  |  |
| **CaHA-CPMB vs. CaHA-CPMV** | **0.2613** | **0.2262 to 0.2965** | **Yes** | ******** | **<0.0001** |
| **CaHA-CPMB vs. CaHA-CPMI** | **0.2117** | **0.1765 to 0.2468** | **Yes** | ******** | **<0.0001** |
| **CaHA-CPMB vs. CaHA-CPMR** | **-0.1197** | **-0.1548 to -0.08453** | **Yes** | ******** | **<0.0001** |
| **CaHA-CPMV vs. CaHA-CPMI** | **-0.04967** | **-0.08480 to -0.01453** | **Yes** | ****** | **0.0025** |
| **CaHA-CPMV vs. CaHA-CPMR** | **-0.381** | **-0.4161 to -0.3459** | **Yes** | ******** | **<0.0001** |
| **CaHA-CPMI vs. CaHA-CPMR** | **-0.3313** | **-0.3665 to -0.2962** | **Yes** | ******** | **<0.0001** |
|  |  |  |  |  |  |
| **1:3** |  |  |  |  |  |
| **CaHA-CPMB vs. CaHA-CPMV** | **0.3633** | **0.3282 to 0.3985** | **Yes** | ******** | **<0.0001** |
| **CaHA-CPMB vs. CaHA-CPMI** | **0.307** | **0.2719 to 0.3421** | **Yes** | ******** | **<0.0001** |
| **CaHA-CPMB vs. CaHA-CPMR** | **-0.08767** | **-0.1228 to -0.05253** | **Yes** | ******** | **<0.0001** |
| **CaHA-CPMV vs. CaHA-CPMI** | **-0.05633** | **-0.09147 to -0.02120** | **Yes** | ******* | **0.0005** |
| **CaHA-CPMV vs. CaHA-CPMR** | **-0.451** | **-0.4861 to -0.4159** | **Yes** | ******** | **<0.0001** |
| **CaHA-CPMI vs. CaHA-CPMR** | **-0.3947** | **-0.4298 to -0.3595** | **Yes** | ******** | **<0.0001** |
|  |  |  |  |  |  |
| **1:4** |  |  |  |  |  |
| **CaHA-CPMB vs. CaHA-CPMV** | **0.3887** | **0.3535 to 0.4238** | **Yes** | ******** | **<0.0001** |
| **CaHA-CPMB vs. CaHA-CPMI** | **0.329** | **0.2939 to 0.3641** | **Yes** | ******** | **<0.0001** |
| **CaHA-CPMB vs. CaHA-CPMR** | **-0.1433** | **-0.1785 to -0.1082** | **Yes** | ******** | **<0.0001** |
| **CaHA-CPMV vs. CaHA-CPMI** | **-0.05967** | **-0.09480 to -0.02453** | **Yes** | ******* | **0.0002** |
| **CaHA-CPMV vs. CaHA-CPMR** | **-0.532** | **-0.5671 to -0.4969** | **Yes** | ******** | **<0.0001** |
| **CaHA-CPMI vs. CaHA-CPMR** | **-0.4723** | **-0.5075 to -0.4372** | **Yes** | ******** | **<0.0001** |
|  |  |  |  |  |  |
| **CPM Only** | |  |  |  |  |
| **CaHA-CPMB vs. CaHA-CPMV** | **0.5787** | **0.5435 to 0.6138** | **Yes** | ******** | **<0.0001** |
| **CaHA-CPMB vs. CaHA-CPMI** | **0.4703** | **0.4352 to 0.5055** | **Yes** | ******** | **<0.0001** |
| **CaHA-CPMB vs. CaHA-CPMR** | **-0.2803** | **-0.3155 to -0.2452** | **Yes** | ******** | **<0.0001** |
| **CaHA-CPMV vs. CaHA-CPMI** | **-0.1083** | **-0.1435 to -0.07320** | **Yes** | ******** | **<0.0001** |
| **CaHA-CPMV vs. CaHA-CPMR** | **-0.859** | **-0.8941 to -0.8239** | **Yes** | ******** | **<0.0001** |
| **CaHA-CPMI vs. CaHA-CPMR** | **-0.7507** | **-0.7858 to -0.7155** | **Yes** | ******** | **<0.0001** |
|  |  |  |  |  |  |
| **CaHA-CPMB** | |  |  |  |  |
| **CaHA-CMC Only vs. 1:1** | **-0.1837** | **-0.2228 to -0.1445** | **Yes** | ******** | **<0.0001** |
| **CaHA-CMC Only vs. 1:2** | **-0.2453** | **-0.2845 to -0.2062** | **Yes** | ******** | **<0.0001** |
| **CaHA-CMC Only vs. 1:3** | **-0.324** | **-0.3632 to -0.2848** | **Yes** | ******** | **<0.0001** |
| **CaHA-CMC Only vs. 1:4** | **-0.3277** | **-0.3668 to -0.2885** | **Yes** | ******** | **<0.0001** |
| **CaHA-CMC Only vs. CPM Only** | **-0.4297** | **-0.4688 to -0.3905** | **Yes** | ******** | **<0.0001** |
| **1:1 vs. 1:2** | **-0.06167** | **-0.1008 to -0.02248** | **Yes** | ******* | **0.0003** |
| **1:1 vs. 1:3** | **-0.1403** | **-0.1795 to -0.1012** | **Yes** | ******** | **<0.0001** |
| **1:1 vs. 1:4** | **-0.144** | **-0.1832 to -0.1048** | **Yes** | ******** | **<0.0001** |
| **1:1 vs. CPM Only** | **-0.246** | **-0.2852 to -0.2068** | **Yes** | ******** | **<0.0001** |
| **1:2 vs. 1:3** | **-0.07867** | **-0.1178 to -0.03948** | **Yes** | ******** | **<0.0001** |
| **1:2 vs. 1:4** | **-0.08233** | **-0.1215 to -0.04315** | **Yes** | ******** | **<0.0001** |
| **1:2 vs. CPM Only** | **-0.1843** | **-0.2235 to -0.1452** | **Yes** | ******** | **<0.0001** |
| **1:3 vs. 1:4** | **-0.00367** | **-0.04285 to 0.03552** | **No** | **ns** | **0.9998** |
| **1:3 vs. CPM Only** | **-0.1057** | **-0.1448 to -0.06648** | **Yes** | ******** | **<0.0001** |
| **1:4 vs. CPM Only** | **-0.102** | **-0.1412 to -0.06282** | **Yes** | ******** | **<0.0001** |
|  |  |  |  |  |  |
| **CaHA-CPMV** | |  |  |  |  |
| **CaHA-CMC Only vs. 1:1** | **-0.037** | **-0.07618 to 0.002183** | **No** | **ns** | **0.0743** |
| **CaHA-CMC Only vs. 1:2** | **0.016** | **-0.02318 to 0.05518** | **No** | **ns** | **0.8289** |
| **CaHA-CMC Only vs. 1:3** | **0.03933** | **0.0001508 to 0.07852** | **Yes** | ***** | **0.0486** |
| **CaHA-CMC Only vs. 1:4** | **0.061** | **0.02182 to 0.1002** | **Yes** | ******* | **0.0004** |
| **CaHA-CMC Only vs. CPM Only** | **0.149** | **0.1098 to 0.1882** | **Yes** | ******** | **<0.0001** |
| **1:1 vs. 1:2** | **0.053** | **0.01382 to 0.09218** | **Yes** | ****** | **0.0027** |
| **1:1 vs. 1:3** | **0.07633** | **0.03715 to 0.1155** | **Yes** | ******** | **<0.0001** |
| **1:1 vs. 1:4** | **0.098** | **0.05882 to 0.1372** | **Yes** | ******** | **<0.0001** |
| **1:1 vs. CPM Only** | **0.186** | **0.1468 to 0.2252** | **Yes** | ******** | **<0.0001** |
| **1:2 vs. 1:3** | **0.02333** | **-0.01585 to 0.06252** | **No** | **ns** | **0.4958** |
| **1:2 vs. 1:4** | **0.045** | **0.005817 to 0.08418** | **Yes** | ***** | **0.0158** |
| **1:2 vs. CPM Only** | **0.133** | **0.09382 to 0.1722** | **Yes** | ******** | **<0.0001** |
| **1:3 vs. 1:4** | **0.02167** | **-0.01752 to 0.06085** | **No** | **ns** | **0.5762** |
| **1:3 vs. CPM Only** | **0.1097** | **0.07048 to 0.1488** | **Yes** | ******** | **<0.0001** |
| **1:4 vs. CPM Only** | **0.088** | **0.04882 to 0.1272** | **Yes** | ******** | **<0.0001** |
|  |  |  |  |  |  |
| **CaHA-CPMI** | |  |  |  |  |
| **CaHA-CMC Only vs. 1:1** | **-0.05433** | **-0.09352 to -0.01515** | **Yes** | ****** | **0.002** |
| **CaHA-CMC Only vs. 1:2** | **-0.03367** | **-0.07285 to 0.005516** | **No** | **ns** | **0.1301** |
| **CaHA-CMC Only vs. 1:3** | **-0.017** | **-0.05618 to 0.02218** | **No** | **ns** | **0.7902** |
| **CaHA-CMC Only vs. 1:4** | **0.001333** | **-0.03785 to 0.04052** | **No** | **ns** | **>0.9999** |
| **CaHA-CMC Only vs. CPM Only** | **0.04067** | **0.001484 to 0.07985** | **Yes** | ***** | **0.0378** |
| **1:1 vs. 1:2** | **0.02067** | **-0.01852 to 0.05985** | **No** | **ns** | **0.6246** |
| **1:1 vs. 1:3** | **0.03733** | **-0.001849 to 0.07652** | **No** | **ns** | **0.07** |
| **1:1 vs. 1:4** | **0.05567** | **0.01648 to 0.09485** | **Yes** | ****** | **0.0014** |
| **1:1 vs. CPM Only** | **0.095** | **0.05582 to 0.1342** | **Yes** | ******** | **<0.0001** |
| **1:2 vs. 1:3** | **0.01667** | **-0.02252 to 0.05585** | **No** | **ns** | **0.8035** |
| **1:2 vs. 1:4** | **0.035** | **-0.004183 to 0.07418** | **No** | **ns** | **0.1047** |
| **1:2 vs. CPM Only** | **0.07433** | **0.03515 to 0.1135** | **Yes** | ******** | **<0.0001** |
| **1:3 vs. 1:4** | **0.01833** | **-0.02085 to 0.05752** | **No** | **ns** | **0.7335** |
| **1:3 vs. CPM Only** | **0.05767** | **0.01848 to 0.09685** | **Yes** | ******* | **0.0009** |
| **1:4 vs. CPM Only** | **0.03933** | **0.0001508 to 0.07852** | **Yes** | ***** | **0.0486** |
|  |  |  |  |  |  |
| **CaHA-CPMR** | |  |  |  |  |
| **CaHA-CMC Only vs. 1:1** | **-0.2813** | **-0.3205 to -0.2422** | **Yes** | ******** | **<0.0001** |
| **CaHA-CMC Only vs. 1:2** | **-0.365** | **-0.4042 to -0.3258** | **Yes** | ******** | **<0.0001** |
| **CaHA-CMC Only vs. 1:3** | **-0.4117** | **-0.4508 to -0.3725** | **Yes** | ******** | **<0.0001** |
| **CaHA-CMC Only vs. 1:4** | **-0.471** | **-0.5102 to -0.4318** | **Yes** | ******** | **<0.0001** |
| **CaHA-CMC Only vs. CPM Only** | **-0.71** | **-0.7492 to -0.6708** | **Yes** | ******** | **<0.0001** |
| **1:1 vs. 1:2** | **-0.08367** | **-0.1228 to -0.04448** | **Yes** | ******** | **<0.0001** |
| **1:1 vs. 1:3** | **-0.1303** | **-0.1695 to -0.09115** | **Yes** | ******** | **<0.0001** |
| **1:1 vs. 1:4** | **-0.1897** | **-0.2288 to -0.1505** | **Yes** | ******** | **<0.0001** |
| **1:1 vs. CPM Only** | **-0.4287** | **-0.4678 to -0.3895** | **Yes** | ******** | **<0.0001** |
| **1:2 vs. 1:3** | **-0.04667** | **-0.08585 to -0.007484** | **Yes** | ***** | **0.0111** |
| **1:2 vs. 1:4** | **-0.106** | **-0.1452 to -0.06682** | **Yes** | ******** | **<0.0001** |
| **1:2 vs. CPM Only** | **-0.345** | **-0.3842 to -0.3058** | **Yes** | ******** | **<0.0001** |
| **1:3 vs. 1:4** | **-0.05933** | **-0.09852 to -0.02015** | **Yes** | ******* | **0.0006** |
| **1:3 vs. CPM Only** | **-0.2983** | **-0.3375 to -0.2592** | **Yes** | ******** | **<0.0001** |
| **1:4 vs. CPM Only** | **-0.239** | **-0.2782 to -0.1998** | **Yes** | ******** | **<0.0001** |

**Table S6. Pairwise comparisons of measured complex moduli (G*).**

| **Tukey's multiple comparisons test** | **Mean Diff.** | **95.00% CI of diff.** | **Below threshold?** | **Summary** | **Adjusted P Value** |
| --- | --- | --- | --- | --- | --- |
|  |  |  |  |  |  |
| **CaHA-CMC Only** | |  |  |  |  |
| **CaHA-CPMB vs. CaHA-CPMV** | **0** | **-59.55 to 59.55** | **No** | **ns** | **>0.9999** |
| **CaHA-CPMB vs. CaHA-CPMI** | **0** | **-59.55 to 59.55** | **No** | **ns** | **>0.9999** |
| **CaHA-CPMB vs. CaHA-CPMR** | **0** | **-59.55 to 59.55** | **No** | **ns** | **>0.9999** |
| **CaHA-CPMV vs. CaHA-CPMI** | **0** | **-59.55 to 59.55** | **No** | **ns** | **>0.9999** |
| **CaHA-CPMV vs. CaHA-CPMR** | **0** | **-59.55 to 59.55** | **No** | **ns** | **>0.9999** |
| **CaHA-CPMI vs. CaHA-CPMR** | **0** | **-59.55 to 59.55** | **No** | **ns** | **>0.9999** |
|  |  |  |  |  |  |
| **1:1** |  |  |  |  |  |
| **CaHA-CPMB vs. CaHA-CPMV** | **-359.2** | **-418.8 to -299.7** | **Yes** | ******** | **<0.0001** |
| **CaHA-CPMB vs. CaHA-CPMI** | **-206.4** | **-266.0 to -146.9** | **Yes** | ******** | **<0.0001** |
| **CaHA-CPMB vs. CaHA-CPMR** | **91.04** | **31.49 to 150.6** | **Yes** | ******* | **0.001** |
| **CaHA-CPMV vs. CaHA-CPMI** | **152.8** | **93.27 to 212.4** | **Yes** | ******** | **<0.0001** |
| **CaHA-CPMV vs. CaHA-CPMR** | **450.3** | **390.7 to 509.8** | **Yes** | ******** | **<0.0001** |
| **CaHA-CPMI vs. CaHA-CPMR** | **297.5** | **237.9 to 357.0** | **Yes** | ******** | **<0.0001** |
|  |  |  |  |  |  |
| **1:2** |  |  |  |  |  |
| **CaHA-CPMB vs. CaHA-CPMV** | **-317** | **-376.6 to -257.5** | **Yes** | ******** | **<0.0001** |
| **CaHA-CPMB vs. CaHA-CPMI** | **-230.1** | **-289.6 to -170.5** | **Yes** | ******** | **<0.0001** |
| **CaHA-CPMB vs. CaHA-CPMR** | **36.75** | **-22.81 to 96.30** | **No** | **ns** | **0.3653** |
| **CaHA-CPMV vs. CaHA-CPMI** | **86.98** | **27.42 to 146.5** | **Yes** | ****** | **0.0017** |
| **CaHA-CPMV vs. CaHA-CPMR** | **353.8** | **294.2 to 413.3** | **Yes** | ******** | **<0.0001** |
| **CaHA-CPMI vs. CaHA-CPMR** | **266.8** | **207.3 to 326.4** | **Yes** | ******** | **<0.0001** |
|  |  |  |  |  |  |
| **1:3** |  |  |  |  |  |
| **CaHA-CPMB vs. CaHA-CPMV** | **-308.7** | **-368.3 to -249.2** | **Yes** | ******** | **<0.0001** |
| **CaHA-CPMB vs. CaHA-CPMI** | **-225.6** | **-285.2 to -166.1** | **Yes** | ******** | **<0.0001** |
| **CaHA-CPMB vs. CaHA-CPMR** | **8.316** | **-51.24 to 67.87** | **No** | **ns** | **0.9823** |
| **CaHA-CPMV vs. CaHA-CPMI** | **83.11** | **23.56 to 142.7** | **Yes** | ****** | **0.0029** |
| **CaHA-CPMV vs. CaHA-CPMR** | **317** | **257.5 to 376.6** | **Yes** | ******** | **<0.0001** |
| **CaHA-CPMI vs. CaHA-CPMR** | **233.9** | **174.4 to 293.5** | **Yes** | ******** | **<0.0001** |
|  |  |  |  |  |  |
| **1:4** |  |  |  |  |  |
| **CaHA-CPMB vs. CaHA-CPMV** | **-286.3** | **-345.9 to -226.8** | **Yes** | ******** | **<0.0001** |
| **CaHA-CPMB vs. CaHA-CPMI** | **-205.7** | **-265.3 to -146.2** | **Yes** | ******** | **<0.0001** |
| **CaHA-CPMB vs. CaHA-CPMR** | **9.1** | **-50.45 to 68.65** | **No** | **ns** | **0.977** |
| **CaHA-CPMV vs. CaHA-CPMI** | **80.56** | **21.00 to 140.1** | **Yes** | ****** | **0.0041** |
| **CaHA-CPMV vs. CaHA-CPMR** | **295.4** | **235.9 to 355.0** | **Yes** | ******** | **<0.0001** |
| **CaHA-CPMI vs. CaHA-CPMR** | **214.8** | **155.3 to 274.4** | **Yes** | ******** | **<0.0001** |
|  |  |  |  |  |  |
| **CPM Only** | |  |  |  |  |
| **CaHA-CPMB vs. CaHA-CPMV** | **-208.7** | **-268.3 to -149.1** | **Yes** | ******** | **<0.0001** |
| **CaHA-CPMB vs. CaHA-CPMI** | **-140.3** | **-199.9 to -80.74** | **Yes** | ******** | **<0.0001** |
| **CaHA-CPMB vs. CaHA-CPMR** | **26.64** | **-32.91 to 86.19** | **No** | **ns** | **0.6357** |
| **CaHA-CPMV vs. CaHA-CPMI** | **68.4** | **8.846 to 128.0** | **Yes** | ***** | **0.0185** |
| **CaHA-CPMV vs. CaHA-CPMR** | **235.3** | **175.8 to 294.9** | **Yes** | ******** | **<0.0001** |
| **CaHA-CPMI vs. CaHA-CPMR** | **166.9** | **107.4 to 226.5** | **Yes** | ******** | **<0.0001** |
|  |  |  |  |  |  |
| **CaHA-CPMB** | |  |  |  |  |
| **CaHA-CMC Only vs. 1:1** | **2020** | **1954 to 2086** | **Yes** | ******** | **<0.0001** |
| **CaHA-CMC Only vs. 1:2** | **2213** | **2146 to 2279** | **Yes** | ******** | **<0.0001** |
| **CaHA-CMC Only vs. 1:3** | **2284** | **2218 to 2350** | **Yes** | ******** | **<0.0001** |
| **CaHA-CMC Only vs. 1:4** | **2302** | **2235 to 2368** | **Yes** | ******** | **<0.0001** |
| **CaHA-CMC Only vs. CPM Only** | **2371** | **2304 to 2437** | **Yes** | ******** | **<0.0001** |
| **1:1 vs. 1:2** | **192.6** | **126.2 to 259.0** | **Yes** | ******** | **<0.0001** |
| **1:1 vs. 1:3** | **264.1** | **197.6 to 330.5** | **Yes** | ******** | **<0.0001** |
| **1:1 vs. 1:4** | **281.5** | **215.1 to 347.9** | **Yes** | ******** | **<0.0001** |
| **1:1 vs. CPM Only** | **350.6** | **284.2 to 417.0** | **Yes** | ******** | **<0.0001** |
| **1:2 vs. 1:3** | **71.47** | **5.054 to 137.9** | **Yes** | ***** | **0.0282** |
| **1:2 vs. 1:4** | **88.94** | **22.52 to 155.3** | **Yes** | ****** | **0.0031** |
| **1:2 vs. CPM Only** | **158** | **91.58 to 224.4** | **Yes** | ******** | **<0.0001** |
| **1:3 vs. 1:4** | **17.47** | **-48.94 to 83.88** | **No** | **ns** | **0.9695** |
| **1:3 vs. CPM Only** | **86.53** | **20.12 to 152.9** | **Yes** | ****** | **0.0042** |
| **1:4 vs. CPM Only** | **69.06** | **2.647 to 135.5** | **Yes** | ***** | **0.0372** |
|  |  |  |  |  |  |
| **CaHA-CPMV** | |  |  |  |  |
| **CaHA-CMC Only vs. 1:1** | **1661** | **1594 to 1727** | **Yes** | ******** | **<0.0001** |
| **CaHA-CMC Only vs. 1:2** | **1896** | **1829 to 1962** | **Yes** | ******** | **<0.0001** |
| **CaHA-CMC Only vs. 1:3** | **1975** | **1909 to 2042** | **Yes** | ******** | **<0.0001** |
| **CaHA-CMC Only vs. 1:4** | **2015** | **1949 to 2082** | **Yes** | ******** | **<0.0001** |
| **CaHA-CMC Only vs. CPM Only** | **2162** | **2095 to 2228** | **Yes** | ******** | **<0.0001** |
| **1:1 vs. 1:2** | **234.8** | **168.4 to 301.2** | **Yes** | ******** | **<0.0001** |
| **1:1 vs. 1:3** | **314.6** | **248.1 to 381.0** | **Yes** | ******** | **<0.0001** |
| **1:1 vs. 1:4** | **354.4** | **288.0 to 420.9** | **Yes** | ******** | **<0.0001** |
| **1:1 vs. CPM Only** | **501.1** | **434.7 to 567.5** | **Yes** | ******** | **<0.0001** |
| **1:2 vs. 1:3** | **79.78** | **13.36 to 146.2** | **Yes** | ***** | **0.0102** |
| **1:2 vs. 1:4** | **119.7** | **53.26 to 186.1** | **Yes** | ******** | **<0.0001** |
| **1:2 vs. CPM Only** | **266.3** | **199.9 to 332.8** | **Yes** | ******** | **<0.0001** |
| **1:3 vs. 1:4** | **39.9** | **-26.51 to 106.3** | **No** | **ns** | **0.486** |
| **1:3 vs. CPM Only** | **186.6** | **120.2 to 253.0** | **Yes** | ******** | **<0.0001** |
| **1:4 vs. CPM Only** | **146.7** | **80.26 to 213.1** | **Yes** | ******** | **<0.0001** |
|  |  |  |  |  |  |
| **CaHA-CPMI** | |  |  |  |  |
| **CaHA-CMC Only vs. 1:1** | **1814** | **1747 to 1880** | **Yes** | ******** | **<0.0001** |
| **CaHA-CMC Only vs. 1:2** | **1983** | **1916 to 2049** | **Yes** | ******** | **<0.0001** |
| **CaHA-CMC Only vs. 1:3** | **2058** | **1992 to 2125** | **Yes** | ******** | **<0.0001** |
| **CaHA-CMC Only vs. 1:4** | **2096** | **2029 to 2162** | **Yes** | ******** | **<0.0001** |
| **CaHA-CMC Only vs. CPM Only** | **2230** | **2164 to 2297** | **Yes** | ******** | **<0.0001** |
| **1:1 vs. 1:2** | **168.9** | **102.5 to 235.3** | **Yes** | ******** | **<0.0001** |
| **1:1 vs. 1:3** | **244.8** | **178.4 to 311.3** | **Yes** | ******** | **<0.0001** |
| **1:1 vs. 1:4** | **282.2** | **215.8 to 348.6** | **Yes** | ******** | **<0.0001** |
| **1:1 vs. CPM Only** | **416.7** | **350.3 to 483.1** | **Yes** | ******** | **<0.0001** |
| **1:2 vs. 1:3** | **75.91** | **9.498 to 142.3** | **Yes** | ***** | **0.0165** |
| **1:2 vs. 1:4** | **113.3** | **46.84 to 179.7** | **Yes** | ******** | **<0.0001** |
| **1:2 vs. CPM Only** | **247.8** | **181.4 to 314.2** | **Yes** | ******** | **<0.0001** |
| **1:3 vs. 1:4** | **37.35** | **-29.07 to 103.8** | **No** | **ns** | **0.5584** |
| **1:3 vs. CPM Only** | **171.9** | **105.4 to 238.3** | **Yes** | ******** | **<0.0001** |
| **1:4 vs. CPM Only** | **134.5** | **68.10 to 200.9** | **Yes** | ******** | **<0.0001** |
|  |  |  |  |  |  |
| **CaHA-CPMR** | |  |  |  |  |
| **CaHA-CMC Only vs. 1:1** | **2111** | **2045 to 2177** | **Yes** | ******** | **<0.0001** |
| **CaHA-CMC Only vs. 1:2** | **2249** | **2183 to 2316** | **Yes** | ******** | **<0.0001** |
| **CaHA-CMC Only vs. 1:3** | **2292** | **2226 to 2359** | **Yes** | ******** | **<0.0001** |
| **CaHA-CMC Only vs. 1:4** | **2311** | **2244 to 2377** | **Yes** | ******** | **<0.0001** |
| **CaHA-CMC Only vs. CPM Only** | **2397** | **2331 to 2464** | **Yes** | ******** | **<0.0001** |
| **1:1 vs. 1:2** | **138.3** | **71.88 to 204.7** | **Yes** | ******** | **<0.0001** |
| **1:1 vs. 1:3** | **181.3** | **114.9 to 247.7** | **Yes** | ******** | **<0.0001** |
| **1:1 vs. 1:4** | **199.6** | **133.2 to 266.0** | **Yes** | ******** | **<0.0001** |
| **1:1 vs. CPM Only** | **286.2** | **219.8 to 352.6** | **Yes** | ******** | **<0.0001** |
| **1:2 vs. 1:3** | **43.03** | **-23.38 to 109.4** | **No** | **ns** | **0.401** |
| **1:2 vs. 1:4** | **61.29** | **-5.123 to 127.7** | **No** | **ns** | **0.086** |
| **1:2 vs. CPM Only** | **147.9** | **81.48 to 214.3** | **Yes** | ******** | **<0.0001** |
| **1:3 vs. 1:4** | **18.25** | **-48.16 to 84.67** | **No** | **ns** | **0.9632** |
| **1:3 vs. CPM Only** | **104.9** | **38.44 to 171.3** | **Yes** | ******* | **0.0003** |
| **1:4 vs. CPM Only** | **86.6** | **20.19 to 153.0** | **Yes** | ****** | **0.0042** |

**Table S7. Pairwise comparisons of measured drop weight values.**

| **Tukey's multiple comparisons test** | **Predicted (LS) mean diff.** | **95.00% CI of diff.** | **Below threshold?** | **Summary** | **Adjusted P Value** |
| --- | --- | --- | --- | --- | --- |
|  |  |  |  |  |  |
| **CaHA-CMC Only** | |  |  |  |  |
| **CaHA-CPMB vs. CaHA-CPMV** | **0** | **-5.915 to 5.915** | **No** | **ns** | **>0.9999** |
| **CaHA-CPMB vs. CaHA-CPMI** | **0** | **-5.915 to 5.915** | **No** | **ns** | **>0.9999** |
| **CaHA-CPMB vs. CaHA-CPMR** | **0** | **-5.915 to 5.915** | **No** | **ns** | **>0.9999** |
| **CaHA-CPMV vs. CaHA-CPMI** | **0** | **-5.915 to 5.915** | **No** | **ns** | **>0.9999** |
| **CaHA-CPMV vs. CaHA-CPMR** | **0** | **-5.915 to 5.915** | **No** | **ns** | **>0.9999** |
| **CaHA-CPMI vs. CaHA-CPMR** | **0** | **-5.915 to 5.915** | **No** | **ns** | **>0.9999** |
|  |  |  |  |  |  |
| **1:1** |  |  |  |  |  |
| **CaHA-CPMB vs. CaHA-CPMV** | **5.942** | **2.527 to 9.357** | **Yes** | ******* | **0.0002** |
| **CaHA-CPMB vs. CaHA-CPMI** | **-4.281** | **-7.696 to -0.8661** | **Yes** | ****** | **0.009** |
| **CaHA-CPMB vs. CaHA-CPMR** | **2.433** | **-0.9815 to 5.848** | **No** | **ns** | **0.2402** |
| **CaHA-CPMV vs. CaHA-CPMI** | **-10.22** | **-13.64 to -6.808** | **Yes** | ******** | **<0.0001** |
| **CaHA-CPMV vs. CaHA-CPMR** | **-3.509** | **-6.924 to -0.09413** | **Yes** | ***** | **0.042** |
| **CaHA-CPMI vs. CaHA-CPMR** | **6.714** | **3.299 to 10.13** | **Yes** | ******** | **<0.0001** |
|  |  |  |  |  |  |
| **1:2** |  |  |  |  |  |
| **CaHA-CPMB vs. CaHA-CPMV** | **7.561** | **4.146 to 10.98** | **Yes** | ******** | **<0.0001** |
| **CaHA-CPMB vs. CaHA-CPMI** | **0.664** | **-2.751 to 4.079** | **No** | **ns** | **0.9535** |
| **CaHA-CPMB vs. CaHA-CPMR** | **5.186** | **1.771 to 8.601** | **Yes** | ****** | **0.0012** |
| **CaHA-CPMV vs. CaHA-CPMI** | **-6.897** | **-10.31 to -3.482** | **Yes** | ******** | **<0.0001** |
| **CaHA-CPMV vs. CaHA-CPMR** | **-2.376** | **-5.791 to 1.039** | **No** | **ns** | **0.2593** |
| **CaHA-CPMI vs. CaHA-CPMR** | **4.522** | **1.107 to 7.937** | **Yes** | ****** | **0.0053** |
|  |  |  |  |  |  |
| **1:3** |  |  |  |  |  |
| **CaHA-CPMB vs. CaHA-CPMV** | **8.381** | **4.966 to 11.80** | **Yes** | ******** | **<0.0001** |
| **CaHA-CPMB vs. CaHA-CPMI** | **1.306** | **-2.109 to 4.721** | **No** | **ns** | **0.7358** |
| **CaHA-CPMB vs. CaHA-CPMR** | **2.422** | **-0.9932 to 5.837** | **No** | **ns** | **0.244** |
| **CaHA-CPMV vs. CaHA-CPMI** | **-7.075** | **-10.49 to -3.660** | **Yes** | ******** | **<0.0001** |
| **CaHA-CPMV vs. CaHA-CPMR** | **-5.959** | **-9.374 to -2.544** | **Yes** | ******* | **0.0002** |
| **CaHA-CPMI vs. CaHA-CPMR** | **1.116** | **-2.299 to 4.531** | **No** | **ns** | **0.8173** |
|  |  |  |  |  |  |
| **1:4** |  |  |  |  |  |
| **CaHA-CPMB vs. CaHA-CPMV** | **8.814** | **5.399 to 12.23** | **Yes** | ******** | **<0.0001** |
| **CaHA-CPMB vs. CaHA-CPMI** | **2.21** | **-1.205 to 5.625** | **No** | **ns** | **0.3196** |
| **CaHA-CPMB vs. CaHA-CPMR** | **2.686** | **-0.7285 to 6.101** | **No** | **ns** | **0.1679** |
| **CaHA-CPMV vs. CaHA-CPMI** | **-6.604** | **-10.02 to -3.189** | **Yes** | ******** | **<0.0001** |
| **CaHA-CPMV vs. CaHA-CPMR** | **-6.128** | **-9.543 to -2.713** | **Yes** | ******* | **0.0001** |
| **CaHA-CPMI vs. CaHA-CPMR** | **0.476** | **-2.939 to 3.891** | **No** | **ns** | **0.982** |
|  |  |  |  |  |  |
| **CPM Only** | |  |  |  |  |
| **CaHA-CPMB vs. CaHA-CPMV** | **35.43** | **32.02 to 38.85** | **Yes** | ******** | **<0.0001** |
| **CaHA-CPMB vs. CaHA-CPMI** | **19.13** | **15.72 to 22.55** | **Yes** | ******** | **<0.0001** |
| **CaHA-CPMB vs. CaHA-CPMR** | **3.7** | **0.2851 to 7.115** | **Yes** | ***** | **0.0292** |
| **CaHA-CPMV vs. CaHA-CPMI** | **-16.3** | **-19.71 to -12.89** | **Yes** | ******** | **<0.0001** |
| **CaHA-CPMV vs. CaHA-CPMR** | **-31.73** | **-35.15 to -28.32** | **Yes** | ******** | **<0.0001** |
| **CaHA-CPMI vs. CaHA-CPMR** | **-15.43** | **-18.85 to -12.02** | **Yes** | ******** | **<0.0001** |
|  |  |  |  |  |  |
| **CaHA-CPMB** | |  |  |  |  |
| **CaHA-CMC Only vs. 1:1** | **19.2** | **13.81 to 24.59** | **Yes** | ******** | **<0.0001** |
| **CaHA-CMC Only vs. 1:2** | **16.56** | **11.17 to 21.95** | **Yes** | ******** | **<0.0001** |
| **CaHA-CMC Only vs. 1:3** | **16.55** | **11.15 to 21.94** | **Yes** | ******** | **<0.0001** |
| **CaHA-CMC Only vs. 1:4** | **15.87** | **10.48 to 21.26** | **Yes** | ******** | **<0.0001** |
| **CaHA-CMC Only vs. CPM Only** | **-10.53** | **-15.92 to -5.142** | **Yes** | ******** | **<0.0001** |
| **1:1 vs. 1:2** | **-2.639** | **-6.451 to 1.173** | **No** | **ns** | **0.3226** |
| **1:1 vs. 1:3** | **-2.652** | **-6.464 to 1.160** | **No** | **ns** | **0.3172** |
| **1:1 vs. 1:4** | **-3.325** | **-7.137 to 0.4868** | **No** | **ns** | **0.1183** |
| **1:1 vs. CPM Only** | **-29.73** | **-33.54 to -25.92** | **Yes** | ******** | **<0.0001** |
| **1:2 vs. 1:3** | **-0.01333** | **-3.825 to 3.799** | **No** | **ns** | **>0.9999** |
| **1:2 vs. 1:4** | **-0.6867** | **-4.499 to 3.125** | **No** | **ns** | **0.9942** |
| **1:2 vs. CPM Only** | **-27.09** | **-30.90 to -23.28** | **Yes** | ******** | **<0.0001** |
| **1:3 vs. 1:4** | **-0.6733** | **-4.485 to 3.139** | **No** | **ns** | **0.9947** |
| **1:3 vs. CPM Only** | **-27.08** | **-30.89 to -23.27** | **Yes** | ******** | **<0.0001** |
| **1:4 vs. CPM Only** | **-26.41** | **-30.22 to -22.59** | **Yes** | ******** | **<0.0001** |
|  |  |  |  |  |  |
| **CaHA-CPMV** | |  |  |  |  |
| **CaHA-CMC Only vs. 1:1** | **25.14** | **19.75 to 30.53** | **Yes** | ******** | **<0.0001** |
| **CaHA-CMC Only vs. 1:2** | **24.12** | **18.73 to 29.51** | **Yes** | ******** | **<0.0001** |
| **CaHA-CMC Only vs. 1:3** | **24.93** | **19.53 to 30.32** | **Yes** | ******** | **<0.0001** |
| **CaHA-CMC Only vs. 1:4** | **24.69** | **19.29 to 30.08** | **Yes** | ******** | **<0.0001** |
| **CaHA-CMC Only vs. CPM Only** | **24.9** | **19.51 to 30.29** | **Yes** | ******** | **<0.0001** |
| **1:1 vs. 1:2** | **-1.02** | **-4.832 to 2.792** | **No** | **ns** | **0.9658** |
| **1:1 vs. 1:3** | **-0.2137** | **-4.026 to 3.598** | **No** | **ns** | **>0.9999** |
| **1:1 vs. 1:4** | **-0.4537** | **-4.266 to 3.358** | **No** | **ns** | **0.9992** |
| **1:1 vs. CPM Only** | **-0.2393** | **-4.051 to 3.573** | **No** | **ns** | **>0.9999** |
| **1:2 vs. 1:3** | **0.806** | **-3.006 to 4.618** | **No** | **ns** | **0.9878** |
| **1:2 vs. 1:4** | **0.566** | **-3.246 to 4.378** | **No** | **ns** | **0.9977** |
| **1:2 vs. CPM Only** | **0.7803** | **-3.032 to 4.592** | **No** | **ns** | **0.9895** |
| **1:3 vs. 1:4** | **-0.24** | **-4.052 to 3.572** | **No** | **ns** | **>0.9999** |
| **1:3 vs. CPM Only** | **-0.02567** | **-3.838 to 3.786** | **No** | **ns** | **>0.9999** |
| **1:4 vs. CPM Only** | **0.2143** | **-3.598 to 4.026** | **No** | **ns** | **>0.9999** |
|  |  |  |  |  |  |
| **CaHA-CPMI** | |  |  |  |  |
| **CaHA-CMC Only vs. 1:1** | **14.92** | **9.525 to 20.31** | **Yes** | ******** | **<0.0001** |
| **CaHA-CMC Only vs. 1:2** | **17.22** | **11.83 to 22.61** | **Yes** | ******** | **<0.0001** |
| **CaHA-CMC Only vs. 1:3** | **17.85** | **12.46 to 23.24** | **Yes** | ******** | **<0.0001** |
| **CaHA-CMC Only vs. 1:4** | **18.08** | **12.69 to 23.47** | **Yes** | ******** | **<0.0001** |
| **CaHA-CMC Only vs. CPM Only** | **8.6** | **3.209 to 13.99** | **Yes** | ******* | **0.0003** |
| **1:1 vs. 1:2** | **2.306** | **-1.506 to 6.118** | **No** | **ns** | **0.4709** |
| **1:1 vs. 1:3** | **2.935** | **-0.8771 to 6.747** | **No** | **ns** | **0.2165** |
| **1:1 vs. 1:4** | **3.166** | **-0.6461 to 6.978** | **No** | **ns** | **0.1529** |
| **1:1 vs. CPM Only** | **-6.316** | **-10.13 to -2.504** | **Yes** | ******* | **0.0002** |
| **1:2 vs. 1:3** | **0.6287** | **-3.183 to 4.441** | **No** | **ns** | **0.9961** |
| **1:2 vs. 1:4** | **0.8597** | **-2.952 to 4.672** | **No** | **ns** | **0.9838** |
| **1:2 vs. CPM Only** | **-8.622** | **-12.43 to -4.810** | **Yes** | ******** | **<0.0001** |
| **1:3 vs. 1:4** | **0.231** | **-3.581 to 4.043** | **No** | **ns** | **>0.9999** |
| **1:3 vs. CPM Only** | **-9.251** | **-13.06 to -5.439** | **Yes** | ******** | **<0.0001** |
| **1:4 vs. CPM Only** | **-9.482** | **-13.29 to -5.670** | **Yes** | ******** | **<0.0001** |
|  |  |  |  |  |  |
| **CaHA-CPMR** | |  |  |  |  |
| **CaHA-CMC Only vs. 1:1** | **21.63** | **16.24 to 27.02** | **Yes** | ******** | **<0.0001** |
| **CaHA-CMC Only vs. 1:2** | **21.74** | **16.35 to 27.14** | **Yes** | ******** | **<0.0001** |
| **CaHA-CMC Only vs. 1:3** | **18.97** | **13.58 to 24.36** | **Yes** | ******** | **<0.0001** |
| **CaHA-CMC Only vs. 1:4** | **18.56** | **13.17 to 23.95** | **Yes** | ******** | **<0.0001** |
| **CaHA-CMC Only vs. CPM Only** | **-6.833** | **-12.22 to -1.442** | **Yes** | ****** | **0.0061** |
| **1:1 vs. 1:2** | **0.1137** | **-3.698 to 3.926** | **No** | **ns** | **>0.9999** |
| **1:1 vs. 1:3** | **-2.664** | **-6.476 to 1.148** | **No** | **ns** | **0.3126** |
| **1:1 vs. 1:4** | **-3.072** | **-6.884 to 0.7398** | **No** | **ns** | **0.1767** |
| **1:1 vs. CPM Only** | **-28.46** | **-32.28 to -24.65** | **Yes** | ******** | **<0.0001** |
| **1:2 vs. 1:3** | **-2.777** | **-6.589 to 1.035** | **No** | **ns** | **0.2695** |
| **1:2 vs. 1:4** | **-3.186** | **-6.998 to 0.6261** | **No** | **ns** | **0.1482** |
| **1:2 vs. CPM Only** | **-28.58** | **-32.39 to -24.77** | **Yes** | ******** | **<0.0001** |
| **1:3 vs. 1:4** | **-0.4087** | **-4.221 to 3.403** | **No** | **ns** | **0.9995** |
| **1:3 vs. CPM Only** | **-25.8** | **-29.61 to -21.99** | **Yes** | ******** | **<0.0001** |
| **1:4 vs. CPM Only** | **-25.39** | **-29.20 to -21.58** | **Yes** | ******** | **<0.0001** |

**Table S8. Pairwise comparisons of measured extrusion forces.**

| **Tukey's multiple comparisons test** | **Mean Diff.** | **95.00% CI of diff.** | **Below threshold?** | **Summary** | **Adjusted P Value** |
| --- | --- | --- | --- | --- | --- |
|  |  |  |  |  |  |
| **CaHA-CMC Only** | |  |  |  |  |
| **CaHA-CPMB vs. CaHA-CPMV** | **0** | **-2.943 to 2.943** | **No** | **ns** | **>0.9999** |
| **CaHA-CPMB vs. CaHA-CPMI** | **0** | **-2.943 to 2.943** | **No** | **ns** | **>0.9999** |
| **CaHA-CPMB vs. CaHA-CPMR** | **0** | **-2.943 to 2.943** | **No** | **ns** | **>0.9999** |
| **CaHA-CPMV vs. CaHA-CPMI** | **0** | **-2.943 to 2.943** | **No** | **ns** | **>0.9999** |
| **CaHA-CPMV vs. CaHA-CPMR** | **0** | **-2.943 to 2.943** | **No** | **ns** | **>0.9999** |
| **CaHA-CPMI vs. CaHA-CPMR** | **0** | **-2.943 to 2.943** | **No** | **ns** | **>0.9999** |
|  |  |  |  |  |  |
| **1:1** |  |  |  |  |  |
| **CaHA-CPMB vs. CaHA-CPMV** | **2.165** | **-0.7783 to 5.108** | **No** | **ns** | **0.2184** |
| **CaHA-CPMB vs. CaHA-CPMI** | **-1.318** | **-4.261 to 1.625** | **No** | **ns** | **0.6349** |
| **CaHA-CPMB vs. CaHA-CPMR** | **4.157** | **1.214 to 7.100** | **Yes** | ****** | **0.0025** |
| **CaHA-CPMV vs. CaHA-CPMI** | **-3.483** | **-6.426 to -0.5397** | **Yes** | ***** | **0.0145** |
| **CaHA-CPMV vs. CaHA-CPMR** | **1.993** | **-0.9503 to 4.936** | **No** | **ns** | **0.285** |
| **CaHA-CPMI vs. CaHA-CPMR** | **5.475** | **2.532 to 8.418** | **Yes** | ******** | **<0.0001** |
|  |  |  |  |  |  |
| **1:2** |  |  |  |  |  |
| **CaHA-CPMB vs. CaHA-CPMV** | **-0.379** | **-3.322 to 2.564** | **No** | **ns** | **0.986** |
| **CaHA-CPMB vs. CaHA-CPMI** | **-3.299** | **-6.242 to -0.3557** | **Yes** | ***** | **0.0225** |
| **CaHA-CPMB vs. CaHA-CPMR** | **3.156** | **0.2134 to 6.099** | **Yes** | ***** | **0.0312** |
| **CaHA-CPMV vs. CaHA-CPMI** | **-2.92** | **-5.863 to 0.02330** | **No** | **ns** | **0.0526** |
| **CaHA-CPMV vs. CaHA-CPMR** | **3.535** | **0.5924 to 6.478** | **Yes** | ***** | **0.0127** |
| **CaHA-CPMI vs. CaHA-CPMR** | **6.455** | **3.512 to 9.398** | **Yes** | ******** | **<0.0001** |
|  |  |  |  |  |  |
| **1:3** |  |  |  |  |  |
| **CaHA-CPMB vs. CaHA-CPMV** | **-1.593** | **-4.536 to 1.350** | **No** | **ns** | **0.4807** |
| **CaHA-CPMB vs. CaHA-CPMI** | **-3.597** | **-6.540 to -0.6537** | **Yes** | ***** | **0.0109** |
| **CaHA-CPMB vs. CaHA-CPMR** | **3.053** | **0.1104 to 5.996** | **Yes** | ***** | **0.0393** |
| **CaHA-CPMV vs. CaHA-CPMI** | **-2.003** | **-4.946 to 0.9396** | **No** | **ns** | **0.2805** |
| **CaHA-CPMV vs. CaHA-CPMR** | **4.647** | **1.704 to 7.590** | **Yes** | ******* | **0.0006** |
| **CaHA-CPMI vs. CaHA-CPMR** | **6.65** | **3.707 to 9.593** | **Yes** | ******** | **<0.0001** |
|  |  |  |  |  |  |
| **1:4** |  |  |  |  |  |
| **CaHA-CPMB vs. CaHA-CPMV** | **-0.5933** | **-3.536 to 2.350** | **No** | **ns** | **0.9497** |
| **CaHA-CPMB vs. CaHA-CPMI** | **-3.707** | **-6.650 to -0.7637** | **Yes** | ****** | **0.0083** |
| **CaHA-CPMB vs. CaHA-CPMR** | **3.85** | **0.9070 to 6.793** | **Yes** | ****** | **0.0057** |
| **CaHA-CPMV vs. CaHA-CPMI** | **-3.113** | **-6.056 to -0.1704** | **Yes** | ***** | **0.0344** |
| **CaHA-CPMV vs. CaHA-CPMR** | **4.443** | **1.500 to 7.386** | **Yes** | ****** | **0.0011** |
| **CaHA-CPMI vs. CaHA-CPMR** | **7.557** | **4.614 to 10.50** | **Yes** | ******** | **<0.0001** |
|  |  |  |  |  |  |
| **CPM Only** | |  |  |  |  |
| **CaHA-CPMB vs. CaHA-CPMV** | **-0.783** | **-3.726 to 2.160** | **No** | **ns** | **0.8934** |
| **CaHA-CPMB vs. CaHA-CPMI** | **-11.74** | **-14.68 to -8.797** | **Yes** | ******** | **<0.0001** |
| **CaHA-CPMB vs. CaHA-CPMR** | **1.254** | **-1.689 to 4.197** | **No** | **ns** | **0.6706** |
| **CaHA-CPMV vs. CaHA-CPMI** | **-10.96** | **-13.90 to -8.014** | **Yes** | ******** | **<0.0001** |
| **CaHA-CPMV vs. CaHA-CPMR** | **2.037** | **-0.9060 to 4.980** | **No** | **ns** | **0.2667** |
| **CaHA-CPMI vs. CaHA-CPMR** | **12.99** | **10.05 to 15.94** | **Yes** | ******** | **<0.0001** |
|  |  |  |  |  |  |
| **CaHA-CPMB** | |  |  |  |  |
| **CaHA-CMC Only vs. 1:1** | **0.602** | **-2.680 to 3.884** | **No** | **ns** | **0.994** |
| **CaHA-CMC Only vs. 1:2** | **2.844** | **-0.4383 to 6.126** | **No** | **ns** | **0.1243** |
| **CaHA-CMC Only vs. 1:3** | **4.01** | **0.7277 to 7.292** | **Yes** | ****** | **0.0086** |
| **CaHA-CMC Only vs. 1:4** | **3.723** | **0.4411 to 7.005** | **Yes** | ***** | **0.0177** |
| **CaHA-CMC Only vs. CPM Only** | **8.294** | **5.012 to 11.58** | **Yes** | ******** | **<0.0001** |
| **1:1 vs. 1:2** | **2.242** | **-1.040 to 5.524** | **No** | **ns** | **0.3426** |
| **1:1 vs. 1:3** | **3.408** | **0.1257 to 6.690** | **Yes** | ***** | **0.0376** |
| **1:1 vs. 1:4** | **3.121** | **-0.1609 to 6.403** | **No** | **ns** | **0.0709** |
| **1:1 vs. CPM Only** | **7.692** | **4.410 to 10.97** | **Yes** | ******** | **<0.0001** |
| **1:2 vs. 1:3** | **1.166** | **-2.116 to 4.448** | **No** | **ns** | **0.8966** |
| **1:2 vs. 1:4** | **0.8793** | **-2.403 to 4.161** | **No** | **ns** | **0.967** |
| **1:2 vs. CPM Only** | **5.45** | **2.168 to 8.732** | **Yes** | ******* | **0.0001** |
| **1:3 vs. 1:4** | **-0.2867** | **-3.569 to 2.995** | **No** | **ns** | **0.9998** |
| **1:3 vs. CPM Only** | **4.284** | **1.002 to 7.566** | **Yes** | ****** | **0.0041** |
| **1:4 vs. CPM Only** | **4.571** | **1.289 to 7.853** | **Yes** | ****** | **0.0019** |
|  |  |  |  |  |  |
| **CaHA-CPMV** | |  |  |  |  |
| **CaHA-CMC Only vs. 1:1** | **2.767** | **-0.5153 to 6.049** | **No** | **ns** | **0.1439** |
| **CaHA-CMC Only vs. 1:2** | **2.465** | **-0.8173 to 5.747** | **No** | **ns** | **0.2439** |
| **CaHA-CMC Only vs. 1:3** | **2.416** | **-0.8656 to 5.698** | **No** | **ns** | **0.2636** |
| **CaHA-CMC Only vs. 1:4** | **3.13** | **-0.1523 to 6.412** | **No** | **ns** | **0.0696** |
| **CaHA-CMC Only vs. CPM Only** | **7.511** | **4.229 to 10.79** | **Yes** | ******** | **<0.0001** |
| **1:1 vs. 1:2** | **-0.302** | **-3.584 to 2.980** | **No** | **ns** | **0.9998** |
| **1:1 vs. 1:3** | **-0.3503** | **-3.632 to 2.932** | **No** | **ns** | **0.9995** |
| **1:1 vs. 1:4** | **0.363** | **-2.919 to 3.645** | **No** | **ns** | **0.9995** |
| **1:1 vs. CPM Only** | **4.744** | **1.462 to 8.026** | **Yes** | ****** | **0.0011** |
| **1:2 vs. 1:3** | **-0.04833** | **-3.330 to 3.234** | **No** | **ns** | **>0.9999** |
| **1:2 vs. 1:4** | **0.665** | **-2.617 to 3.947** | **No** | **ns** | **0.9904** |
| **1:2 vs. CPM Only** | **5.046** | **1.764 to 8.328** | **Yes** | ******* | **0.0005** |
| **1:3 vs. 1:4** | **0.7133** | **-2.569 to 3.995** | **No** | **ns** | **0.9869** |
| **1:3 vs. CPM Only** | **5.094** | **1.812 to 8.376** | **Yes** | ******* | **0.0004** |
| **1:4 vs. CPM Only** | **4.381** | **1.099 to 7.663** | **Yes** | ****** | **0.0032** |
|  |  |  |  |  |  |
| **CaHA-CPMI** | |  |  |  |  |
| **CaHA-CMC Only vs. 1:1** | **-0.716** | **-3.998 to 2.566** | **No** | **ns** | **0.9866** |
| **CaHA-CMC Only vs. 1:2** | **-0.455** | **-3.737 to 2.827** | **No** | **ns** | **0.9984** |
| **CaHA-CMC Only vs. 1:3** | **0.413** | **-2.869 to 3.695** | **No** | **ns** | **0.999** |
| **CaHA-CMC Only vs. 1:4** | **0.01633** | **-3.266 to 3.298** | **No** | **ns** | **>0.9999** |
| **CaHA-CMC Only vs. CPM Only** | **-3.447** | **-6.729 to -0.1647** | **Yes** | ***** | **0.0344** |
| **1:1 vs. 1:2** | **0.261** | **-3.021 to 3.543** | **No** | **ns** | **0.9999** |
| **1:1 vs. 1:3** | **1.129** | **-2.153 to 4.411** | **No** | **ns** | **0.9086** |
| **1:1 vs. 1:4** | **0.7323** | **-2.550 to 4.014** | **No** | **ns** | **0.9852** |
| **1:1 vs. CPM Only** | **-2.731** | **-6.013 to 0.5513** | **No** | **ns** | **0.1538** |
| **1:2 vs. 1:3** | **0.868** | **-2.414 to 4.150** | **No** | **ns** | **0.9688** |
| **1:2 vs. 1:4** | **0.4713** | **-2.811 to 3.753** | **No** | **ns** | **0.9981** |
| **1:2 vs. CPM Only** | **-2.992** | **-6.274 to 0.2903** | **No** | **ns** | **0.0928** |
| **1:3 vs. 1:4** | **-0.3967** | **-3.679 to 2.885** | **No** | **ns** | **0.9992** |
| **1:3 vs. CPM Only** | **-3.86** | **-7.142 to -0.5777** | **Yes** | ***** | **0.0126** |
| **1:4 vs. CPM Only** | **-3.463** | **-6.745 to -0.1811** | **Yes** | ***** | **0.0331** |
|  |  |  |  |  |  |
| **CaHA-CPMR** | |  |  |  |  |
| **CaHA-CMC Only vs. 1:1** | **4.759** | **1.477 to 8.041** | **Yes** | ****** | **0.0011** |
| **CaHA-CMC Only vs. 1:2** | **6** | **2.718 to 9.282** | **Yes** | ******** | **<0.0001** |
| **CaHA-CMC Only vs. 1:3** | **7.063** | **3.781 to 10.34** | **Yes** | ******** | **<0.0001** |
| **CaHA-CMC Only vs. 1:4** | **7.573** | **4.291 to 10.85** | **Yes** | ******** | **<0.0001** |
| **CaHA-CMC Only vs. CPM Only** | **9.548** | **6.266 to 12.83** | **Yes** | ******** | **<0.0001** |
| **1:1 vs. 1:2** | **1.241** | **-2.041 to 4.523** | **No** | **ns** | **0.8699** |
| **1:1 vs. 1:3** | **2.304** | **-0.9783 to 5.586** | **No** | **ns** | **0.3131** |
| **1:1 vs. 1:4** | **2.814** | **-0.4683 to 6.096** | **No** | **ns** | **0.1317** |
| **1:1 vs. CPM Only** | **4.788** | **1.506 to 8.070** | **Yes** | ****** | **0.001** |
| **1:2 vs. 1:3** | **1.063** | **-2.219 to 4.345** | **No** | **ns** | **0.9278** |
| **1:2 vs. 1:4** | **1.573** | **-1.709 to 4.855** | **No** | **ns** | **0.7134** |
| **1:2 vs. CPM Only** | **3.548** | **0.2657 to 6.830** | **Yes** | ***** | **0.0271** |
| **1:3 vs. 1:4** | **0.51** | **-2.772 to 3.792** | **No** | **ns** | **0.9972** |
| **1:3 vs. CPM Only** | **2.485** | **-0.7973 to 5.767** | **No** | **ns** | **0.2361** |
| **1:4 vs. CPM Only** | **1.975** | **-1.307 to 5.257** | **No** | **ns** | **0.4843** |

**Table S9. Pairwise comparisons of measured axial strains.**

| **Tukey's multiple comparisons test** | **Mean Diff.** | **95.00% CI of diff.** | **Below threshold?** | **Summary** | **Adjusted P Value** |
| --- | --- | --- | --- | --- | --- |
|  |  |  |  |  |  |
| **CaHA-CMC Only** | |  |  |  |  |
| **CaHA-CPMB vs. CaHA-CPMV** | **0** | **-0.03383 to 0.03383** | **No** | **ns** | **>0.9999** |
| **CaHA-CPMB vs. CaHA-CPMI** | **0** | **-0.03383 to 0.03383** | **No** | **ns** | **>0.9999** |
| **CaHA-CPMB vs. CaHA-CPMR** | **0** | **-0.03383 to 0.03383** | **No** | **ns** | **>0.9999** |
| **CaHA-CPMV vs. CaHA-CPMI** | **0** | **-0.03383 to 0.03383** | **No** | **ns** | **>0.9999** |
| **CaHA-CPMV vs. CaHA-CPMR** | **0** | **-0.03383 to 0.03383** | **No** | **ns** | **>0.9999** |
| **CaHA-CPMI vs. CaHA-CPMR** | **0** | **-0.03383 to 0.03383** | **No** | **ns** | **>0.9999** |
|  |  |  |  |  |  |
| **1:1** |  |  |  |  |  |
| **CaHA-CPMB vs. CaHA-CPMV** | **0.106** | **0.07217 to 0.1398** | **Yes** | ******** | **<0.0001** |
| **CaHA-CPMB vs. CaHA-CPMI** | **0.09367** | **0.05984 to 0.1275** | **Yes** | ******** | **<0.0001** |
| **CaHA-CPMB vs. CaHA-CPMR** | **-0.043** | **-0.07683 to -0.009171** | **Yes** | ****** | **0.0076** |
| **CaHA-CPMV vs. CaHA-CPMI** | **-0.01233** | **-0.04616 to 0.02150** | **No** | **ns** | **0.7669** |
| **CaHA-CPMV vs. CaHA-CPMR** | **-0.149** | **-0.1828 to -0.1152** | **Yes** | ******** | **<0.0001** |
| **CaHA-CPMI vs. CaHA-CPMR** | **-0.1367** | **-0.1705 to -0.1028** | **Yes** | ******** | **<0.0001** |
|  |  |  |  |  |  |
| **1:2** |  |  |  |  |  |
| **CaHA-CPMB vs. CaHA-CPMV** | **0.23** | **0.1962 to 0.2638** | **Yes** | ******** | **<0.0001** |
| **CaHA-CPMB vs. CaHA-CPMI** | **0.222** | **0.1882 to 0.2558** | **Yes** | ******** | **<0.0001** |
| **CaHA-CPMB vs. CaHA-CPMR** | **-0.05733** | **-0.09116 to -0.02350** | **Yes** | ******* | **0.0002** |
| **CaHA-CPMV vs. CaHA-CPMI** | **-0.008** | **-0.04183 to 0.02583** | **No** | **ns** | **0.9221** |
| **CaHA-CPMV vs. CaHA-CPMR** | **-0.2873** | **-0.3212 to -0.2535** | **Yes** | ******** | **<0.0001** |
| **CaHA-CPMI vs. CaHA-CPMR** | **-0.2793** | **-0.3132 to -0.2455** | **Yes** | ******** | **<0.0001** |
|  |  |  |  |  |  |
| **1:3** |  |  |  |  |  |
| **CaHA-CPMB vs. CaHA-CPMV** | **0.2813** | **0.2475 to 0.3152** | **Yes** | ******** | **<0.0001** |
| **CaHA-CPMB vs. CaHA-CPMI** | **0.2627** | **0.2288 to 0.2965** | **Yes** | ******** | **<0.0001** |
| **CaHA-CPMB vs. CaHA-CPMR** | **-0.08633** | **-0.1202 to -0.05250** | **Yes** | ******** | **<0.0001** |
| **CaHA-CPMV vs. CaHA-CPMI** | **-0.01867** | **-0.05250 to 0.01516** | **No** | **ns** | **0.4641** |
| **CaHA-CPMV vs. CaHA-CPMR** | **-0.3677** | **-0.4015 to -0.3338** | **Yes** | ******** | **<0.0001** |
| **CaHA-CPMI vs. CaHA-CPMR** | **-0.349** | **-0.3828 to -0.3152** | **Yes** | ******** | **<0.0001** |
|  |  |  |  |  |  |
| **1:4** |  |  |  |  |  |
| **CaHA-CPMB vs. CaHA-CPMV** | **0.3133** | **0.2795 to 0.3472** | **Yes** | ******** | **<0.0001** |
| **CaHA-CPMB vs. CaHA-CPMI** | **0.2893** | **0.2555 to 0.3232** | **Yes** | ******** | **<0.0001** |
| **CaHA-CPMB vs. CaHA-CPMR** | **-0.106** | **-0.1398 to -0.07217** | **Yes** | ******** | **<0.0001** |
| **CaHA-CPMV vs. CaHA-CPMI** | **-0.024** | **-0.05783 to 0.009829** | **No** | **ns** | **0.2467** |
| **CaHA-CPMV vs. CaHA-CPMR** | **-0.4193** | **-0.4532 to -0.3855** | **Yes** | ******** | **<0.0001** |
| **CaHA-CPMI vs. CaHA-CPMR** | **-0.3953** | **-0.4292 to -0.3615** | **Yes** | ******** | **<0.0001** |
|  |  |  |  |  |  |
| **CPM Only** | |  |  |  |  |
| **CaHA-CPMB vs. CaHA-CPMV** | **0.4317** | **0.3978 to 0.4655** | **Yes** | ******** | **<0.0001** |
| **CaHA-CPMB vs. CaHA-CPMI** | **0.406** | **0.3722 to 0.4398** | **Yes** | ******** | **<0.0001** |
| **CaHA-CPMB vs. CaHA-CPMR** | **-0.1067** | **-0.1405 to -0.07284** | **Yes** | ******** | **<0.0001** |
| **CaHA-CPMV vs. CaHA-CPMI** | **-0.02567** | **-0.05950 to 0.008163** | **No** | **ns** | **0.1953** |
| **CaHA-CPMV vs. CaHA-CPMR** | **-0.5383** | **-0.5722 to -0.5045** | **Yes** | ******** | **<0.0001** |
| **CaHA-CPMI vs. CaHA-CPMR** | **-0.5127** | **-0.5465 to -0.4788** | **Yes** | ******** | **<0.0001** |
|  |  |  |  |  |  |
| **CaHA-CPMB** | |  |  |  |  |
| **CaHA-CMC Only vs. 1:1** | **-0.139** | **-0.1767 to -0.1013** | **Yes** | ******** | **<0.0001** |
| **CaHA-CMC Only vs. 1:2** | **-0.279** | **-0.3167 to -0.2413** | **Yes** | ******** | **<0.0001** |
| **CaHA-CMC Only vs. 1:3** | **-0.3397** | **-0.3774 to -0.3019** | **Yes** | ******** | **<0.0001** |
| **CaHA-CMC Only vs. 1:4** | **-0.3747** | **-0.4124 to -0.3369** | **Yes** | ******** | **<0.0001** |
| **CaHA-CMC Only vs. CPM Only** | **-0.5827** | **-0.6204 to -0.5449** | **Yes** | ******** | **<0.0001** |
| **1:1 vs. 1:2** | **-0.14** | **-0.1777 to -0.1023** | **Yes** | ******** | **<0.0001** |
| **1:1 vs. 1:3** | **-0.2007** | **-0.2384 to -0.1629** | **Yes** | ******** | **<0.0001** |
| **1:1 vs. 1:4** | **-0.2357** | **-0.2734 to -0.1979** | **Yes** | ******** | **<0.0001** |
| **1:1 vs. CPM Only** | **-0.4437** | **-0.4814 to -0.4059** | **Yes** | ******** | **<0.0001** |
| **1:2 vs. 1:3** | **-0.06067** | **-0.09839 to -0.02294** | **Yes** | ******* | **0.0002** |
| **1:2 vs. 1:4** | **-0.09567** | **-0.1334 to -0.05794** | **Yes** | ******** | **<0.0001** |
| **1:2 vs. CPM Only** | **-0.3037** | **-0.3414 to -0.2659** | **Yes** | ******** | **<0.0001** |
| **1:3 vs. 1:4** | **-0.035** | **-0.07273 to 0.002725** | **No** | **ns** | **0.0832** |
| **1:3 vs. CPM Only** | **-0.243** | **-0.2807 to -0.2053** | **Yes** | ******** | **<0.0001** |
| **1:4 vs. CPM Only** | **-0.208** | **-0.2457 to -0.1703** | **Yes** | ******** | **<0.0001** |
|  |  |  |  |  |  |
| **CaHA-CPMV** | |  |  |  |  |
| **CaHA-CMC Only vs. 1:1** | **-0.033** | **-0.07073 to 0.004725** | **No** | **ns** | **0.118** |
| **CaHA-CMC Only vs. 1:2** | **-0.049** | **-0.08673 to -0.01127** | **Yes** | ****** | **0.0044** |
| **CaHA-CMC Only vs. 1:3** | **-0.05833** | **-0.09606 to -0.02061** | **Yes** | ******* | **0.0004** |
| **CaHA-CMC Only vs. 1:4** | **-0.06133** | **-0.09906 to -0.02361** | **Yes** | ******* | **0.0002** |
| **CaHA-CMC Only vs. CPM Only** | **-0.151** | **-0.1887 to -0.1133** | **Yes** | ******** | **<0.0001** |
| **1:1 vs. 1:2** | **-0.016** | **-0.05373 to 0.02173** | **No** | **ns** | **0.8054** |
| **1:1 vs. 1:3** | **-0.02533** | **-0.06306 to 0.01239** | **No** | **ns** | **0.3613** |
| **1:1 vs. 1:4** | **-0.02833** | **-0.06606 to 0.009392** | **No** | **ns** | **0.2439** |
| **1:1 vs. CPM Only** | **-0.118** | **-0.1557 to -0.08027** | **Yes** | ******** | **<0.0001** |
| **1:2 vs. 1:3** | **-0.00933** | **-0.04706 to 0.02839** | **No** | **ns** | **0.9766** |
| **1:2 vs. 1:4** | **-0.01233** | **-0.05006 to 0.02539** | **No** | **ns** | **0.9251** |
| **1:2 vs. CPM Only** | **-0.102** | **-0.1397 to -0.06427** | **Yes** | ******** | **<0.0001** |
| **1:3 vs. 1:4** | **-0.003** | **-0.04073 to 0.03473** | **No** | **ns** | **0.9999** |
| **1:3 vs. CPM Only** | **-0.09267** | **-0.1304 to -0.05494** | **Yes** | ******** | **<0.0001** |
| **1:4 vs. CPM Only** | **-0.08967** | **-0.1274 to -0.05194** | **Yes** | ******** | **<0.0001** |
|  |  |  |  |  |  |
| **CaHA-CPMI** | |  |  |  |  |
| **CaHA-CMC Only vs. 1:1** | **-0.04533** | **-0.08306 to -0.007608** | **Yes** | ***** | **0.0102** |
| **CaHA-CMC Only vs. 1:2** | **-0.057** | **-0.09473 to -0.01927** | **Yes** | ******* | **0.0006** |
| **CaHA-CMC Only vs. 1:3** | **-0.077** | **-0.1147 to -0.03927** | **Yes** | ******** | **<0.0001** |
| **CaHA-CMC Only vs. 1:4** | **-0.08533** | **-0.1231 to -0.04761** | **Yes** | ******** | **<0.0001** |
| **CaHA-CMC Only vs. CPM Only** | **-0.1767** | **-0.2144 to -0.1389** | **Yes** | ******** | **<0.0001** |
| **1:1 vs. 1:2** | **-0.01167** | **-0.04939 to 0.02606** | **No** | **ns** | **0.94** |
| **1:1 vs. 1:3** | **-0.03167** | **-0.06939 to 0.006059** | **No** | **ns** | **0.1471** |
| **1:1 vs. 1:4** | **-0.04** | **-0.07773 to -0.002275** | **Yes** | ***** | **0.0318** |
| **1:1 vs. CPM Only** | **-0.1313** | **-0.1691 to -0.09361** | **Yes** | ******** | **<0.0001** |
| **1:2 vs. 1:3** | **-0.02** | **-0.05773 to 0.01773** | **No** | **ns** | **0.6195** |
| **1:2 vs. 1:4** | **-0.02833** | **-0.06606 to 0.009392** | **No** | **ns** | **0.2439** |
| **1:2 vs. CPM Only** | **-0.1197** | **-0.1574 to -0.08194** | **Yes** | ******** | **<0.0001** |
| **1:3 vs. 1:4** | **-0.00833** | **-0.04606 to 0.02939** | **No** | **ns** | **0.9859** |
| **1:3 vs. CPM Only** | **-0.09967** | **-0.1374 to -0.06194** | **Yes** | ******** | **<0.0001** |
| **1:4 vs. CPM Only** | **-0.09133** | **-0.1291 to -0.05361** | **Yes** | ******** | **<0.0001** |
|  |  |  |  |  |  |
| **CaHA-CPMR** | |  |  |  |  |
| **CaHA-CMC Only vs. 1:1** | **-0.182** | **-0.2197 to -0.1443** | **Yes** | ******** | **<0.0001** |
| **CaHA-CMC Only vs. 1:2** | **-0.3363** | **-0.3741 to -0.2986** | **Yes** | ******** | **<0.0001** |
| **CaHA-CMC Only vs. 1:3** | **-0.426** | **-0.4637 to -0.3883** | **Yes** | ******** | **<0.0001** |
| **CaHA-CMC Only vs. 1:4** | **-0.4807** | **-0.5184 to -0.4429** | **Yes** | ******** | **<0.0001** |
| **CaHA-CMC Only vs. CPM Only** | **-0.6893** | **-0.7271 to -0.6516** | **Yes** | ******** | **<0.0001** |
| **1:1 vs. 1:2** | **-0.1543** | **-0.1921 to -0.1166** | **Yes** | ******** | **<0.0001** |
| **1:1 vs. 1:3** | **-0.244** | **-0.2817 to -0.2063** | **Yes** | ******** | **<0.0001** |
| **1:1 vs. 1:4** | **-0.2987** | **-0.3364 to -0.2609** | **Yes** | ******** | **<0.0001** |
| **1:1 vs. CPM Only** | **-0.5073** | **-0.5451 to -0.4696** | **Yes** | ******** | **<0.0001** |
| **1:2 vs. 1:3** | **-0.08967** | **-0.1274 to -0.05194** | **Yes** | ******** | **<0.0001** |
| **1:2 vs. 1:4** | **-0.1443** | **-0.1821 to -0.1066** | **Yes** | ******** | **<0.0001** |
| **1:2 vs. CPM Only** | **-0.353** | **-0.3907 to -0.3153** | **Yes** | ******** | **<0.0001** |
| **1:3 vs. 1:4** | **-0.05467** | **-0.09239 to -0.01694** | **Yes** | ****** | **0.0011** |
| **1:3 vs. CPM Only** | **-0.2633** | **-0.3011 to -0.2256** | **Yes** | ******** | **<0.0001** |
| **1:4 vs. CPM Only** | **-0.2087** | **-0.2464 to -0.1709** | **Yes** | ******** | **<0.0001** |

**Table S10. Pairwise comparisons of CaHA-CPM-V stability over a 24 h period.**

| **Tukey's multiple comparisons test** | **Mean Diff.** | **95.00% CI of diff.** | **Below threshold?** | **Summary** | **Adjusted P Value** |
| --- | --- | --- | --- | --- | --- |
|  |  |  |  |  |  |
| **1:1** |  |  |  |  |  |
| **Immediately vs. 24 h** | **0.1067** | **-0.5544 to 0.7677** | **No** | **ns** | **0.7367** |
|  |  |  |  |  |  |
| **1:2** |  |  |  |  |  |
| **Immediately vs. 24 h** | **-0.4733** | **-1.134 to 0.1877** | **No** | **ns** | **0.1485** |
|  |  |  |  |  |  |
| **1:3** |  |  |  |  |  |
| **Immediately vs. 24 h** | **-0.9233** | **-1.584 to -0.2623** | **Yes** | ****** | **0.0092** |
|  |  |  |  |  |  |
| **1:4** |  |  |  |  |  |
| **Immediately vs. 24 h** | **-0.2667** | **-0.9277 to 0.3944** | **No** | **ns** | **0.4051** |
|  |  |  |  |  |  |
| **Immediately** | |  |  |  |  |
| **1:1 vs. 1:2** | **-0.3767** | **-1.269 to 0.5155** | **No** | **ns** | **0.6308** |
| **1:1 vs. 1:3** | **0.21** | **-0.6821 to 1.102** | **No** | **ns** | **0.9056** |
| **1:1 vs. 1:4** | **0.3833** | **-0.5088 to 1.275** | **No** | **ns** | **0.6181** |
| **1:2 vs. 1:3** | **0.5867** | **-0.3055 to 1.479** | **No** | **ns** | **0.2744** |
| **1:2 vs. 1:4** | **0.76** | **-0.1321 to 1.652** | **No** | **ns** | **0.1097** |
| **1:3 vs. 1:4** | **0.1733** | **-0.7188 to 1.065** | **No** | **ns** | **0.9436** |
|  |  |  |  |  |  |
| **24 h** |  |  |  |  |  |
| **1:1 vs. 1:2** | **-0.9567** | **-1.849 to -0.06455** | **Yes** | ***** | **0.0335** |
| **1:1 vs. 1:3** | **-0.82** | **-1.712 to 0.07212** | **No** | **ns** | **0.0773** |
| **1:1 vs. 1:4** | **0.01** | **-0.8821 to 0.9021** | **No** | **ns** | **>0.9999** |
| **1:2 vs. 1:3** | **0.1367** | **-0.7555 to 1.029** | **No** | **ns** | **0.9709** |
| **1:2 vs. 1:4** | **0.9667** | **0.07455 to 1.859** | **Yes** | ***** | **0.0314** |
| **1:3 vs. 1:4** | **0.83** | **-0.06212 to 1.722** | **No** | **ns** | **0.0729** |

**Table S11. Pairwise comparisons of CaHA-CPM-I stability over a 24 h period.**

| **Tukey's multiple comparisons test** | **Mean Diff.** | **95.00% CI of diff.** | **Below threshold?** | **Summary** | **Adjusted P Value** |
| --- | --- | --- | --- | --- | --- |
|  |  |  |  |  |  |
| **1:1** |  |  |  |  |  |
| **Immediately vs. 24 h** | **0.2033** | **-0.2990 to 0.7057** | **No** | **ns** | **0.4035** |
|  |  |  |  |  |  |
| **1:2** |  |  |  |  |  |
| **Immediately vs. 24 h** | **-0.17** | **-0.6724 to 0.3324** | **No** | **ns** | **0.4835** |
|  |  |  |  |  |  |
| **1:3** |  |  |  |  |  |
| **Immediately vs. 24 h** | **0.04667** | **-0.4557 to 0.5490** | **No** | **ns** | **0.8464** |
|  |  |  |  |  |  |
| **1:4** |  |  |  |  |  |
| **Immediately vs. 24 h** | **0.06** | **-0.4424 to 0.5624** | **No** | **ns** | **0.8033** |
|  |  |  |  |  |  |
| **Immediately** | |  |  |  |  |
| **1:1 vs. 1:2** | **0.3233** | **-0.3546 to 1.001** | **No** | **ns** | **0.5379** |
| **1:1 vs. 1:3** | **0.5767** | **-0.1013 to 1.255** | **No** | **ns** | **0.1105** |
| **1:1 vs. 1:4** | **0.3733** | **-0.3046 to 1.051** | **No** | **ns** | **0.4192** |
| **1:2 vs. 1:3** | **0.2533** | **-0.4246 to 0.9313** | **No** | **ns** | **0.7125** |
| **1:2 vs. 1:4** | **0.05** | **-0.6280 to 0.7280** | **No** | **ns** | **0.9965** |
| **1:3 vs. 1:4** | **-0.2033** | **-0.8813 to 0.4746** | **No** | **ns** | **0.826** |
|  |  |  |  |  |  |
| **24 h** |  |  |  |  |  |
| **1:1 vs. 1:2** | **-0.05** | **-0.7280 to 0.6280** | **No** | **ns** | **0.9965** |
| **1:1 vs. 1:3** | **0.42** | **-0.2580 to 1.098** | **No** | **ns** | **0.3216** |
| **1:1 vs. 1:4** | **0.23** | **-0.4480 to 0.9080** | **No** | **ns** | **0.7677** |
| **1:2 vs. 1:3** | **0.47** | **-0.2080 to 1.148** | **No** | **ns** | **0.2349** |
| **1:2 vs. 1:4** | **0.28** | **-0.3980 to 0.9580** | **No** | **ns** | **0.6465** |
| **1:3 vs. 1:4** | **-0.19** | **-0.8680 to 0.4880** | **No** | **ns** | **0.8526** |

**Table S12. Pairwise comparisons of CaHA-CPM-B stability over a 24 h period.**

| **Tukey's multiple comparisons test** | **Mean Diff.** | **95.00% CI of diff.** | **Below threshold?** | **Summary** | **Adjusted P Value** |
| --- | --- | --- | --- | --- | --- |
|  |  |  |  |  |  |
| **1:1** |  |  |  |  |  |
| **Immediately vs. 24 h** | **0.6467** | **-0.9457 to 2.239** | **No** | **ns** | **0.402** |
|  |  |  |  |  |  |
| **1:2** |  |  |  |  |  |
| **Immediately vs. 24 h** | **-2.247** | **-3.839 to -0.6543** | **Yes** | ****** | **0.0086** |
|  |  |  |  |  |  |
| **1:3** |  |  |  |  |  |
| **Immediately vs. 24 h** | **0.02** | **-1.572 to 1.612** | **No** | **ns** | **0.9791** |
|  |  |  |  |  |  |
| **1:4** |  |  |  |  |  |
| **Immediately vs. 24 h** | **-0.4** | **-1.992 to 1.192** | **No** | **ns** | **0.6017** |
|  |  |  |  |  |  |
| **Immediately** | |  |  |  |  |
| **1:1 vs. 1:2** | **0.9833** | **-1.166 to 3.132** | **No** | **ns** | **0.5706** |
| **1:1 vs. 1:3** | **1.327** | **-0.8224 to 3.476** | **No** | **ns** | **0.3244** |
| **1:1 vs. 1:4** | **1.32** | **-0.8291 to 3.469** | **No** | **ns** | **0.3285** |
| **1:2 vs. 1:3** | **0.3433** | **-1.806 to 2.492** | **No** | **ns** | **0.9673** |
| **1:2 vs. 1:4** | **0.3367** | **-1.812 to 2.486** | **No** | **ns** | **0.969** |
| **1:3 vs. 1:4** | **-0.00667** | **-2.156 to 2.142** | **No** | **ns** | **>0.9999** |
|  |  |  |  |  |  |
| **24 h** |  |  |  |  |  |
| **1:1 vs. 1:2** | **-1.91** | **-4.059 to 0.2391** | **No** | **ns** | **0.0907** |
| **1:1 vs. 1:3** | **0.7** | **-1.449 to 2.849** | **No** | **ns** | **0.7884** |
| **1:1 vs. 1:4** | **0.2733** | **-1.876 to 2.422** | **No** | **ns** | **0.9829** |
| **1:2 vs. 1:3** | **2.61** | **0.4609 to 4.759** | **Yes** | ***** | **0.0149** |
| **1:2 vs. 1:4** | **2.183** | **0.03422 to 4.332** | **Yes** | ***** | **0.0458** |
| **1:3 vs. 1:4** | **-0.4267** | **-2.576 to 1.722** | **No** | **ns** | **0.9402** |

**Table S13. Pairwise comparisons of CaHA-CPM-R stability over a 24 h period.**

| **Tukey's multiple comparisons test** | **Mean Diff.** | **95.00% CI of diff.** | **Below threshold?** | **Summary** | **Adjusted P Value** |
| --- | --- | --- | --- | --- | --- |
|  |  |  |  |  |  |
| **1:1** |  |  |  |  |  |
| **Immediately vs. 24 h** | **-1.743** | **-2.742 to -0.7445** | **Yes** | ****** | **0.0019** |
|  |  |  |  |  |  |
| **1:2** |  |  |  |  |  |
| **Immediately vs. 24 h** | **-0.1133** | **-1.112 to 0.8855** | **No** | **ns** | **0.813** |
|  |  |  |  |  |  |
| **1:3** |  |  |  |  |  |
| **Immediately vs. 24 h** | **-0.1267** | **-1.126 to 0.8722** | **No** | **ns** | **0.7915** |
|  |  |  |  |  |  |
| **1:4** |  |  |  |  |  |
| **Immediately vs. 24 h** | **0.1833** | **-0.8155 to 1.182** | **No** | **ns** | **0.7023** |
|  |  |  |  |  |  |
| **Immediately** | |  |  |  |  |
| **1:1 vs. 1:2** | **0.4633** | **-0.8848 to 1.811** | **No** | **ns** | **0.7608** |
| **1:1 vs. 1:3** | **0.5533** | **-0.7948 to 1.901** | **No** | **ns** | **0.6508** |
| **1:1 vs. 1:4** | **0.1967** | **-1.151 to 1.545** | **No** | **ns** | **0.9747** |
| **1:2 vs. 1:3** | **0.09** | **-1.258 to 1.438** | **No** | **ns** | **0.9974** |
| **1:2 vs. 1:4** | **-0.2667** | **-1.615 to 1.081** | **No** | **ns** | **0.9407** |
| **1:3 vs. 1:4** | **-0.3567** | **-1.705 to 0.9914** | **No** | **ns** | **0.8724** |
|  |  |  |  |  |  |
| **24 h** |  |  |  |  |  |
| **1:1 vs. 1:2** | **2.093** | **0.7452 to 3.441** | **Yes** | ****** | **0.0021** |
| **1:1 vs. 1:3** | **2.17** | **0.8219 to 3.518** | **Yes** | ****** | **0.0015** |
| **1:1 vs. 1:4** | **2.123** | **0.7752 to 3.471** | **Yes** | ****** | **0.0018** |
| **1:2 vs. 1:3** | **0.07667** | **-1.271 to 1.425** | **No** | **ns** | **0.9984** |
| **1:2 vs. 1:4** | **0.03** | **-1.318 to 1.378** | **No** | **ns** | **>0.9999** |
| **1:3 vs. 1:4** | **-0.04667** | **-1.395 to 1.301** | **No** | **ns** | **0.9996** |

**Table S14. Pairwise comparisons of all hybrid stability immediately after preparation.**

| **Tukey's multiple comparisons test** | **Mean Diff.** | **95.00% CI of diff.** | **Below threshold?** | **Summary** | **Adjusted P Value** |
| --- | --- | --- | --- | --- | --- |
|  |  |  |  |  |  |
| **1:1** |  |  |  |  |  |
| **CaHA-CPMV vs. CaHA-CPMI** | **-0.1567** | **-0.7443 to 0.4310** | **No** | **ns** | **0.8875** |
| **CaHA-CPMV vs. CaHA-CPMB** | **-1.033** | **-1.621 to -0.4457** | **Yes** | ******* | **0.0002** |
| **CaHA-CPMV vs. CaHA-CPMR** | **-0.3033** | **-0.8910 to 0.2843** | **No** | **ns** | **0.5094** |
| **CaHA-CPMI vs. CaHA-CPMB** | **-0.8767** | **-1.464 to -0.2890** | **Yes** | ****** | **0.0017** |
| **CaHA-CPMI vs. CaHA-CPMR** | **-0.1467** | **-0.7343 to 0.4410** | **No** | **ns** | **0.9053** |
| **CaHA-CPMB vs. CaHA-CPMR** | **0.73** | **0.1424 to 1.318** | **Yes** | ***** | **0.0102** |
|  |  |  |  |  |  |
| **1:2** |  |  |  |  |  |
| **CaHA-CPMV vs. CaHA-CPMI** | **0.5433** | **-0.04429 to 1.131** | **No** | **ns** | **0.0782** |
| **CaHA-CPMV vs. CaHA-CPMB** | **0.3267** | **-0.2610 to 0.9143** | **No** | **ns** | **0.4457** |
| **CaHA-CPMV vs. CaHA-CPMR** | **0.5367** | **-0.05096 to 1.124** | **No** | **ns** | **0.0835** |
| **CaHA-CPMI vs. CaHA-CPMB** | **-0.2167** | **-0.8043 to 0.3710** | **No** | **ns** | **0.751** |
| **CaHA-CPMI vs. CaHA-CPMR** | **-0.00667** | **-0.5943 to 0.5810** | **No** | **ns** | **>0.9999** |
| **CaHA-CPMB vs. CaHA-CPMR** | **0.21** | **-0.3776 to 0.7976** | **No** | **ns** | **0.7682** |
|  |  |  |  |  |  |
| **1:3** |  |  |  |  |  |
| **CaHA-CPMV vs. CaHA-CPMI** | **0.21** | **-0.3776 to 0.7976** | **No** | **ns** | **0.7682** |
| **CaHA-CPMV vs. CaHA-CPMB** | **0.08333** | **-0.5043 to 0.6710** | **No** | **ns** | **0.9804** |
| **CaHA-CPMV vs. CaHA-CPMR** | **0.04** | **-0.5476 to 0.6276** | **No** | **ns** | **0.9977** |
| **CaHA-CPMI vs. CaHA-CPMB** | **-0.1267** | **-0.7143 to 0.4610** | **No** | **ns** | **0.9362** |
| **CaHA-CPMI vs. CaHA-CPMR** | **-0.17** | **-0.7576 to 0.4176** | **No** | **ns** | **0.8612** |
| **CaHA-CPMB vs. CaHA-CPMR** | **-0.04333** | **-0.6310 to 0.5443** | **No** | **ns** | **0.9971** |
|  |  |  |  |  |  |
| **1:4** |  |  |  |  |  |
| **CaHA-CPMV vs. CaHA-CPMI** | **-0.1667** | **-0.7543 to 0.4210** | **No** | **ns** | **0.868** |
| **CaHA-CPMV vs. CaHA-CPMB** | **-0.09667** | **-0.6843 to 0.4910** | **No** | **ns** | **0.97** |
| **CaHA-CPMV vs. CaHA-CPMR** | **-0.49** | **-1.078 to 0.09763** | **No** | **ns** | **0.1293** |
| **CaHA-CPMI vs. CaHA-CPMB** | **0.07** | **-0.5176 to 0.6576** | **No** | **ns** | **0.9882** |
| **CaHA-CPMI vs. CaHA-CPMR** | **-0.3233** | **-0.9110 to 0.2643** | **No** | **ns** | **0.4547** |
| **CaHA-CPMB vs. CaHA-CPMR** | **-0.3933** | **-0.9810 to 0.1943** | **No** | **ns** | **0.2858** |
|  |  |  |  |  |  |
| **CaHA-CPMV** | |  |  |  |  |
| **1:1 vs. 1:2** | **-0.3767** | **-0.9643 to 0.2110** | **No** | **ns** | **0.3221** |
| **1:1 vs. 1:3** | **0.21** | **-0.3776 to 0.7976** | **No** | **ns** | **0.7682** |
| **1:1 vs. 1:4** | **0.3833** | **-0.2043 to 0.9710** | **No** | **ns** | **0.3072** |
| **1:2 vs. 1:3** | **0.5867** | **-0.0009606 to 1.174** | **No** | **ns** | **0.0505** |
| **1:2 vs. 1:4** | **0.76** | **0.1724 to 1.348** | **Yes** | ****** | **0.0072** |
| **1:3 vs. 1:4** | **0.1733** | **-0.4143 to 0.7610** | **No** | **ns** | **0.8543** |
|  |  |  |  |  |  |
| **CaHA-CPMI** | |  |  |  |  |
| **1:1 vs. 1:2** | **0.3233** | **-0.2643 to 0.9110** | **No** | **ns** | **0.4547** |
| **1:1 vs. 1:3** | **0.5767** | **-0.01096 to 1.164** | **No** | **ns** | **0.056** |
| **1:1 vs. 1:4** | **0.3733** | **-0.2143 to 0.9610** | **No** | **ns** | **0.3296** |
| **1:2 vs. 1:3** | **0.2533** | **-0.3343 to 0.8410** | **No** | **ns** | **0.6509** |
| **1:2 vs. 1:4** | **0.05** | **-0.5376 to 0.6376** | **No** | **ns** | **0.9956** |
| **1:3 vs. 1:4** | **-0.2033** | **-0.7910 to 0.3843** | **No** | **ns** | **0.785** |
|  |  |  |  |  |  |
| **CaHA-CPMB** | |  |  |  |  |
| **1:1 vs. 1:2** | **0.9833** | **0.3957 to 1.571** | **Yes** | ******* | **0.0004** |
| **1:1 vs. 1:3** | **1.327** | **0.7390 to 1.914** | **Yes** | ******** | **<0.0001** |
| **1:1 vs. 1:4** | **1.32** | **0.7324 to 1.908** | **Yes** | ******** | **<0.0001** |
| **1:2 vs. 1:3** | **0.3433** | **-0.2443 to 0.9310** | **No** | **ns** | **0.4023** |
| **1:2 vs. 1:4** | **0.3367** | **-0.2510 to 0.9243** | **No** | **ns** | **0.4194** |
| **1:3 vs. 1:4** | **-0.00667** | **-0.5943 to 0.5810** | **No** | **ns** | **>0.9999** |
|  |  |  |  |  |  |
| **CaHA-CPMR** | |  |  |  |  |
| **1:1 vs. 1:2** | **0.4633** | **-0.1243 to 1.051** | **No** | **ns** | **0.1635** |
| **1:1 vs. 1:3** | **0.5533** | **-0.03429 to 1.141** | **No** | **ns** | **0.0708** |
| **1:1 vs. 1:4** | **0.1967** | **-0.3910 to 0.7843** | **No** | **ns** | **0.8013** |
| **1:2 vs. 1:3** | **0.09** | **-0.4976 to 0.6776** | **No** | **ns** | **0.9755** |
| **1:2 vs. 1:4** | **-0.2667** | **-0.8543 to 0.3210** | **No** | **ns** | **0.6131** |
| **1:3 vs. 1:4** | **-0.3567** | **-0.9443 to 0.2310** | **No** | **ns** | **0.3691** |

**Table S15. Pairwise comparisons of all hybrid stability 24 h after preparation.**

| **Tukey's multiple comparisons test** | **Mean Diff.** | **95.00% CI of diff.** | **Below threshold?** | **Summary** | **Adjusted P Value** |
| --- | --- | --- | --- | --- | --- |
|  |  |  |  |  |  |
| **1:1** |  |  |  |  |  |
| **CaHA-CPMV vs. CaHA-CPMI** | **-0.06** | **-1.822 to 1.702** | **No** | **ns** | **0.9997** |
| **CaHA-CPMV vs. CaHA-CPMB** | **-0.4933** | **-2.255 to 1.268** | **No** | **ns** | **0.8723** |
| **CaHA-CPMV vs. CaHA-CPMR** | **-2.153** | **-3.915 to -0.3916** | **Yes** | ***** | **0.0118** |
| **CaHA-CPMI vs. CaHA-CPMB** | **-0.4333** | **-2.195 to 1.328** | **No** | **ns** | **0.9089** |
| **CaHA-CPMI vs. CaHA-CPMR** | **-2.093** | **-3.855 to -0.3316** | **Yes** | ***** | **0.0148** |
| **CaHA-CPMB vs. CaHA-CPMR** | **-1.66** | **-3.422 to 0.1017** | **No** | **ns** | **0.0706** |
|  |  |  |  |  |  |
| **1:2** |  |  |  |  |  |
| **CaHA-CPMV vs. CaHA-CPMI** | **0.8467** | **-0.9150 to 2.608** | **No** | **ns** | **0.5683** |
| **CaHA-CPMV vs. CaHA-CPMB** | **-1.447** | **-3.208 to 0.3150** | **No** | **ns** | **0.1382** |
| **CaHA-CPMV vs. CaHA-CPMR** | **0.8967** | **-0.8650 to 2.658** | **No** | **ns** | **0.5213** |
| **CaHA-CPMI vs. CaHA-CPMB** | **-2.293** | **-4.055 to -0.5316** | **Yes** | ****** | **0.0067** |
| **CaHA-CPMI vs. CaHA-CPMR** | **0.05** | **-1.712 to 1.812** | **No** | **ns** | **0.9998** |
| **CaHA-CPMB vs. CaHA-CPMR** | **2.343** | **0.5816 to 4.105** | **Yes** | ****** | **0.0055** |
|  |  |  |  |  |  |
| **1:3** |  |  |  |  |  |
| **CaHA-CPMV vs. CaHA-CPMI** | **1.18** | **-0.5817 to 2.942** | **No** | **ns** | **0.2852** |
| **CaHA-CPMV vs. CaHA-CPMB** | **1.027** | **-0.7350 to 2.788** | **No** | **ns** | **0.4045** |
| **CaHA-CPMV vs. CaHA-CPMR** | **0.8367** | **-0.9250 to 2.598** | **No** | **ns** | **0.5778** |
| **CaHA-CPMI vs. CaHA-CPMB** | **-0.1533** | **-1.915 to 1.608** | **No** | **ns** | **0.9953** |
| **CaHA-CPMI vs. CaHA-CPMR** | **-0.3433** | **-2.105 to 1.418** | **No** | **ns** | **0.9517** |
| **CaHA-CPMB vs. CaHA-CPMR** | **-0.19** | **-1.952 to 1.572** | **No** | **ns** | **0.9911** |
|  |  |  |  |  |  |
| **1:4** |  |  |  |  |  |
| **CaHA-CPMV vs. CaHA-CPMI** | **0.16** | **-1.602 to 1.922** | **No** | **ns** | **0.9947** |
| **CaHA-CPMV vs. CaHA-CPMB** | **-0.23** | **-1.992 to 1.532** | **No** | **ns** | **0.9845** |
| **CaHA-CPMV vs. CaHA-CPMR** | **-0.04** | **-1.802 to 1.722** | **No** | **ns** | **>0.9999** |
| **CaHA-CPMI vs. CaHA-CPMB** | **-0.39** | **-2.152 to 1.372** | **No** | **ns** | **0.9314** |
| **CaHA-CPMI vs. CaHA-CPMR** | **-0.2** | **-1.962 to 1.562** | **No** | **ns** | **0.9897** |
| **CaHA-CPMB vs. CaHA-CPMR** | **0.19** | **-1.572 to 1.952** | **No** | **ns** | **0.9911** |
|  |  |  |  |  |  |
| **CaHA-CPMV** | |  |  |  |  |
| **1:1 vs. 1:2** | **-0.9567** | **-2.718 to 0.8050** | **No** | **ns** | **0.4661** |
| **1:1 vs. 1:3** | **-0.82** | **-2.582 to 0.9417** | **No** | **ns** | **0.5936** |
| **1:1 vs. 1:4** | **0.01** | **-1.752 to 1.772** | **No** | **ns** | **>0.9999** |
| **1:2 vs. 1:3** | **0.1367** | **-1.625 to 1.898** | **No** | **ns** | **0.9966** |
| **1:2 vs. 1:4** | **0.9667** | **-0.7950 to 2.728** | **No** | **ns** | **0.4571** |
| **1:3 vs. 1:4** | **0.83** | **-0.9317 to 2.592** | **No** | **ns** | **0.5841** |
|  |  |  |  |  |  |
| **CaHA-CPMI** | |  |  |  |  |
| **1:1 vs. 1:2** | **-0.05** | **-1.812 to 1.712** | **No** | **ns** | **0.9998** |
| **1:1 vs. 1:3** | **0.42** | **-1.342 to 2.182** | **No** | **ns** | **0.9162** |
| **1:1 vs. 1:4** | **0.23** | **-1.532 to 1.992** | **No** | **ns** | **0.9845** |
| **1:2 vs. 1:3** | **0.47** | **-1.292 to 2.232** | **No** | **ns** | **0.8873** |
| **1:2 vs. 1:4** | **0.28** | **-1.482 to 2.042** | **No** | **ns** | **0.9728** |
| **1:3 vs. 1:4** | **-0.19** | **-1.952 to 1.572** | **No** | **ns** | **0.9911** |
|  |  |  |  |  |  |
| **CaHA-CPMB** | |  |  |  |  |
| **1:1 vs. 1:2** | **-1.91** | **-3.672 to -0.1483** | **Yes** | ***** | **0.0295** |
| **1:1 vs. 1:3** | **0.7** | **-1.062 to 2.462** | **No** | **ns** | **0.7061** |
| **1:1 vs. 1:4** | **0.2733** | **-1.488 to 2.035** | **No** | **ns** | **0.9746** |
| **1:2 vs. 1:3** | **2.61** | **0.8483 to 4.372** | **Yes** | ****** | **0.0018** |
| **1:2 vs. 1:4** | **2.183** | **0.4216 to 3.945** | **Yes** | ***** | **0.0104** |
| **1:3 vs. 1:4** | **-0.4267** | **-2.188 to 1.335** | **No** | **ns** | **0.9126** |
|  |  |  |  |  |  |
| **CaHA-CPMR** | |  |  |  |  |
| **1:1 vs. 1:2** | **2.093** | **0.3316 to 3.855** | **Yes** | ***** | **0.0148** |
| **1:1 vs. 1:3** | **2.17** | **0.4083 to 3.932** | **Yes** | ***** | **0.011** |
| **1:1 vs. 1:4** | **2.123** | **0.3616 to 3.885** | **Yes** | ***** | **0.0132** |
| **1:2 vs. 1:3** | **0.07667** | **-1.685 to 1.838** | **No** | **ns** | **0.9994** |
| **1:2 vs. 1:4** | **0.03** | **-1.732 to 1.792** | **No** | **ns** | **>0.9999** |
| **1:3 vs. 1:4** | **-0.04667** | **-1.808 to 1.715** | **No** | **ns** | **0.9999** |

**Table S16. Pairwise comparisons of biostimulatory data derived from Yutskovskaya et al.^[7]^**

| **Tukey's multiple comparisons test** | **Predicted (LS) mean diff.** | **95.00% CI of diff.** | **Below threshold?** | **Summary** | **Adjusted P Value** |
| --- | --- | --- | --- | --- | --- |
|  |  |  |  |  |  |
| **Baseline** |  |  |  |  |  |
| **CaHA-CPMV vs. CaHA-CPMI** | **-16.02** | **-26.45 to -5.584** | **Yes** | ****** | **0.0013** |
| **CaHA-CPMV vs. CaHA-CPMB** | **2.507** | **-7.927 to 12.94** | **No** | **ns** | **0.8344** |
| **CaHA-CPMI vs. CaHA-CPMB** | **18.52** | **8.091 to 28.96** | **Yes** | ******* | **0.0002** |
|  |  |  |  |  |  |
| **1 Month** |  |  |  |  |  |
| **CaHA-CPMV vs. CaHA-CPMI** | **1.446** | **-8.988 to 11.88** | **No** | **ns** | **0.9414** |
| **CaHA-CPMV vs. CaHA-CPMB** | **3.844** | **-6.589 to 14.28** | **No** | **ns** | **0.6544** |
| **CaHA-CPMI vs. CaHA-CPMB** | **2.398** | **-8.036 to 12.83** | **No** | **ns** | **0.8473** |
|  |  |  |  |  |  |
| **4 Months** | |  |  |  |  |
| **CaHA-CPMV vs. CaHA-CPMI** | **14.8** | **3.731 to 25.86** | **Yes** | ****** | **0.0057** |
| **CaHA-CPMV vs. CaHA-CPMB** | **-0.3742** | **-10.81 to 10.06** | **No** | **ns** | **0.996** |
| **CaHA-CPMI vs. CaHA-CPMB** | **-15.17** | **-26.24 to -4.105** | **Yes** | ****** | **0.0044** |
|  |  |  |  |  |  |
| **CaHA-CPMV** | |  |  |  |  |
| **Baseline vs. 1 Month** | **-36.69** | **-47.12 to -26.25** | **Yes** | ******** | **<0.0001** |
| **Baseline vs. 4 Months** | **-41.59** | **-52.03 to -31.16** | **Yes** | ******** | **<0.0001** |
| **1 Month vs. 4 Months** | **-4.906** | **-15.34 to 5.528** | **No** | **ns** | **0.5028** |
|  |  |  |  |  |  |
| **CaHA-CPMI** | |  |  |  |  |
| **Baseline vs. 1 Month** | **-19.22** | **-29.66 to -8.788** | **Yes** | ******** | **<0.0001** |
| **Baseline vs. 4 Months** | **-10.78** | **-21.84 to 0.2903** | **No** | **ns** | **0.058** |
| **1 Month vs. 4 Months** | **8.446** | **-2.621 to 19.51** | **No** | **ns** | **0.1687** |
|  |  |  |  |  |  |
| **CaHA-CPMB** | |  |  |  |  |
| **Baseline vs. 1 Month** | **-35.35** | **-45.78 to -24.91** | **Yes** | ******** | **<0.0001** |
| **Baseline vs. 4 Months** | **-44.47** | **-54.91 to -34.04** | **Yes** | ******** | **<0.0001** |
| **1 Month vs. 4 Months** | **-9.124** | **-19.56 to 1.310** | **No** | **ns** | **0.0987** |
